# Sound identity is represented robustly in auditory cortex during perceptual constancy

**DOI:** 10.1038/s41467-018-07237-3

**Published:** 2018-11-14

**Authors:** Stephen M. Town, Katherine C. Wood, Jennifer K. Bizley

**Affiliations:** 1 Ear Institute, University College London, 332 Gray’s Inn Road, London, WC1X 8EE UK; 2 Present Address: Department of Otorhinolaryngology: HNS, Department of Neuroscience, University of Pennsylvania, Philadelphia, 19104 PA USA

## Abstract

Perceptual constancy requires neural representations that are selective for object identity, but also tolerant across identity-preserving transformations. How such representations arise in the brain and support perception remains unclear. Here, we study tolerant representation of sound identity in the auditory system by recording neural activity in auditory cortex of ferrets during perceptual constancy. Ferrets generalize vowel identity across variations in fundamental frequency, sound level and location, while neurons represent sound identity robustly across acoustic variations. Stimulus features are encoded with distinct time-courses in all conditions, however encoding of sound identity is delayed when animals fail to generalize and during passive listening. Neurons also encode information about task-irrelevant sound features, as well as animals’ choices and accuracy, while population decoding out-performs animals’ behavior. Our results show that during perceptual constancy, sound identity is represented robustly in auditory cortex across widely varying conditions, and behavioral generalization requires conserved timing of identity information.

## Abstract (web summary)

Perceptual constancy requires neural representations selective for object identity, yet tolerant of identity-preserving transformations. Here, the authors show that sound identity is represented robustly in auditory cortex and that behavioral generalization requires precise timing of identity information.

## Introduction

Perceptual constancy, also known as perceptual invariance, is the ability to recognize objects across variations in sensory input, such as a face from multiple angles, or a word spoken by different talkers^1,2^. Perceptual constancy requires that sensory systems, including vision and hearing, develop a level of tolerance to identity-preserving transformations^3,4^. In hearing, tolerance is critical for representing sounds such as individual words or phonemes across talkers, voice pitch, background noise and other acoustic transformations^5^, and is a key step in auditory object formation and scene analysis^1,6,7^.

Both humans and other animals perceive sound features constantly despite variation in sensory input: we can recognize loudness across variation in location^8^, frequency across sound level^9^ and sound identity across talkers^10,11^, vocal tract length^12–14^ and fundamental frequency (F0)^15–17^. At the neural level, tolerance is observed within auditory cortex, where neurons remain informative about the identity of vocalizations^18–20^, pure tones^21^ and pulse trains^22^ across variations in acoustic properties. For speech sounds such as vowels, multiple sound features including phoneme identity, location and F0 modulate activity of auditory cortical neurons^23–26^. However, tolerance has yet to be shown in subjects actively demonstrating perceptual constancy, and the behavioral relevance of previously demonstrated tolerant representations in auditory cortex remains unclear. Furthermore, although auditory cortical processing is modulated by attention and experience^27^, it is unknown how these processes affect tolerant representations.

Here, we asked if tolerant representations for complex sounds exist in early auditory cortex during perceptual constancy, how tolerance was related to behavior, and how tolerance was modulated by attention and experience. To address these questions, we recorded auditory cortical neurons in ferrets discriminating synthesized vowel sounds that varied across identity-preserving acoustic transformations including F0, sound location, level, and voicing. These features varied independently and thus represented orthogonal dimensions in feature space.

We hypothesized that neurons would show tolerance (remain informative about vowel identity) across the same range of orthogonal variables over which animals demonstrate perceptual constancy, and that such tolerance would be degraded if subjects failed to generalize vowel identity. As auditory cortex represents multiple stimulus variables, in the cases where animals generalized successfully, we expected tolerance would exist for both task-relevant and irrelevant sound features. Finally, we predicted that neural correlates of perceptual constancy should be dependent on animals’ behavioral performance, attentional state and training. We found that tolerant representations of sound identity exist during perceptual constancy, and that the timing (but not quantity) of information about vowel identity is associated with behavioral performance. However, the ability of auditory cortex to represent vowel identity exceeds animals’ behavior, and requires neither training, nor task engagement.

## Results

### Perceptual constancy during vowel discrimination

**Fig. 1:**
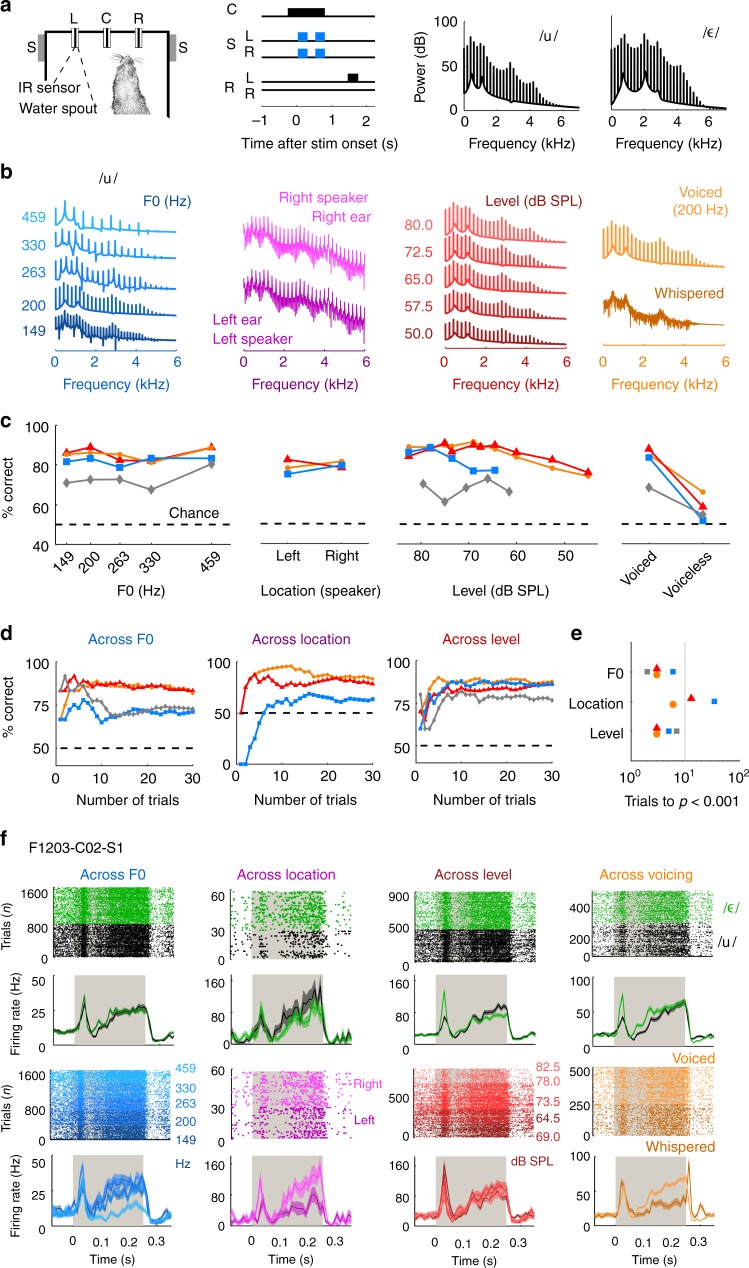
Perceptual constancy during vowel discrimination. **a** Schematic of task design: Animals initiated trials by visiting a central port (C) and waiting for a variable period before stimulus presentation. Speakers (S) presented sounds (two tokens of the same vowel; blue) to the left and right of the head in all conditions except when sounds varied across location—in which case they were presented from either left (S_L_) or right (S_R_) speaker only. Animals responded at the left (R_L_) or right (R_R_) port depending on vowel identity. **b** Spectra for 13 examples of one vowel /u/ with varying F0, location, sound level or voicing. To illustrate the effect of spatial location, the spectra were generated in virtual acoustic space^67^, although sounds presented experimentally varied in their free-field location. **c** Behavioral performance when discriminating vowels across F0, location, level and voicing. Data are shown as performance correct across all test sessions, for each subject separately (F1201: orange circles, F1203: red triangles; 1217; blue squares; F1304: gray diamonds). See Supplementary Table 1 for sample sizes. **d** Performance of subjects as a function of experience. Each line indicates one ferret as in **c**. Dashed lines for **c** and **d** show chance performance (50%). **e** Number of trials required to detect significant task performance when compared to chance (permutation test, *p* < 0.001). Symbols show individual ferrets as in **c**. **f** Raster and peri-stimulus time histograms (PSTHs) of neural responses of one multi-unit to vowels (/u/: black; /ε/: green) across variation in F0 (blue), location (magneta), level (red), and voicing (orange). Data plotted during presentation of the first sound token (gray bar) by vowel identity and by each orthogonal variable. PSTHs show mean ± s.e.m. firing rate across trials

To establish a behavioral model of perceptual constancy, ferrets were trained in a two-choice task (Fig. 1a) to identify synthesized vowels. Once animals were trained, vowels were varied in F0 (149 to 459 Hz), location (±90°), sound level (45 to 82.5 dB Sound Pressure Level [SPL]), or voicing (where vowels were generated to sound whispered and presented on 10 to 20% of trials as probe trials). Changes in these task-irrelevant, orthogonal dimensions produced different spectra while preserving the formant peaks in the spectral envelope (Fig. 1b) critical for vowel identification^28^. On each trial, the animal triggered stimulus presentation consisting of two tokens of the same vowel (250 ms duration, 250 ms interval). Subjects then responded to the left or right depending on vowel identity, with correct responses rewarded with water, and errors triggering brief timeouts (1 to 5 s). Repeated vowel presentation was not necessary for task performance (Supplementary Fig. 1) but was used for consistency with earlier work^15,17^. In each test session, vowels varied across only one orthogonal dimension (e.g., F0). Variation in each orthogonal dimension was sufficient that, had the animals been discriminating these features, performance would have approached ceiling^23,29–32^. Here, behavioral training was required to access subject’s perception, but perceptual constancy itself may occur naturally in ferrets’ perception of sound timbre^33^.

Ferrets discriminated vowels accurately across F0, location and sound level—but not voicing (Fig. 1c). For all F0s, locations and levels, performance was significantly above chance for all subjects, while only two subjects discriminated voiceless vowels successfully (binomial test vs. 50%, *p* < 0.001, Supplementary Table 1). There was no effect of F0 or location on performance (logistic regression, *p* *>* 0.05, Supplementary Table 2), but performance improved significantly, if modestly, with level (3/4 ferrets, logistic regression, *p* < 0.01; Supplementary Table 2). Nevertheless, performance was constant over a range of intensities, and performance at the lowest sound levels still exceeded chance. For all subjects, performance was significantly worse for whispered than voiced vowels. Failure to generalize across voicing may result from delivery of whispered vowels as probe stimuli on 10 to 20% of trials, with any response rewarded. Thus, animals did not receive the same feedback for whispered sounds as for other orthogonal variables. Nonetheless, whispered sounds were presented at equivalent rates as each F0 or sound level (conditions where constancy occurred). Our data may, therefore, reflect limits of ferrets' perceptual constancy resulting from large acoustic differences between voiced and voiceless sounds.

Ferrets accurately reported vowel identity across variations in F0, sound location and sound level. We next asked if vowel discrimination across orthogonal features reflected memorization of correct responses to each sound, or true generalization of sound identity across acoustic input. To test generalization, we calculated animals’ performance as a function of experience: Ferrets learned the original stimulus–response contingency with a single F0, level, location and voicing over thousands of trials. Therefore, if ferrets memorized each stimulus–response association, it should take hundreds of trials to successfully discriminate novel vowels. However, ferrets discriminated vowels with new F0s, sound levels or locations accurately within ten presentations (Fig. 1d and Supplementary Fig. 2). On this time-scale, performance increased with trials, but saturated 10 to 20 trials after introduction; i.e., much faster than the initial discrimination was learnt. Thus, animals rapidly generalized vowel identity to new sounds, arguing against memorization of specific stimulus–response associations. These results are consistent with vowel discrimination across many F0s (*n* = 15) for which memorization is increasingly difficult^17^, and the conclusion that animals perceived a constant sound identity across acoustic variations. We then moved on to ask how neurons in auditory cortex recorded during task performance (Fig. 1f) represented vowels during behavior.

### Decoding acoustic features from neural activity

We implanted microelectrode arrays bilaterally in auditory cortex, where electrodes targeted the low-frequency reversal between tonotopic primary and posterior fields^34,35^. We recorded 502 sound-responsive units (141 single units) and, for each unit, measured responses to vowels across F0, location, level and/or voicing during task performance (Fig. 1f). For some units, activity was recorded in all conditions; however most were tested in a subset of conditions.

We quantified the information available about each sound feature by decoding feature values in one dimension across changes in the orthogonal dimension from single trial unit responses. Our decoder compared the Euclidean distances of time-varying patterns of neural activity, with leave-one-out cross-validation^36^ (Supplementary Fig. 3). We varied the time window over which responses were decoded, and optimized parameters (start time and window duration) in order to compare the timing of information about different sound features (Supplementary Figs. 4–6).

**Fig. 2:**
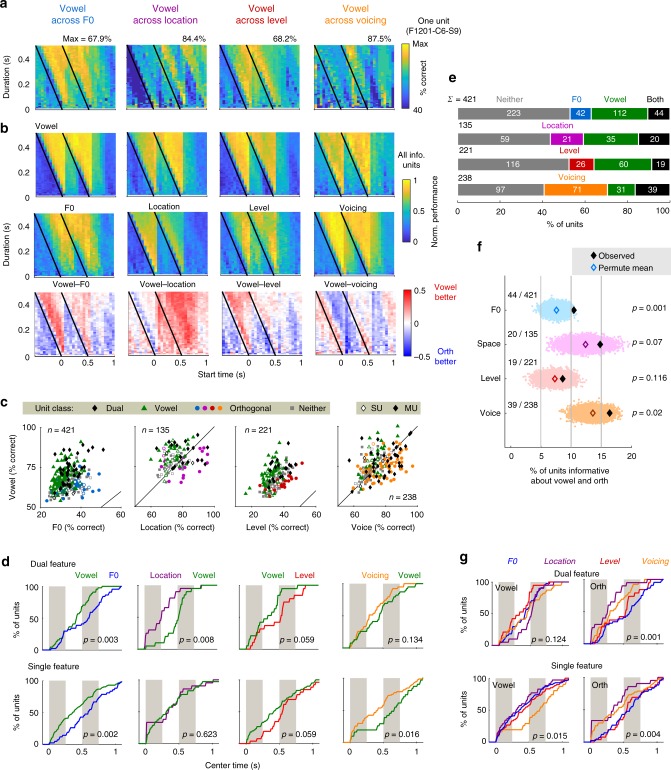
Neural responses and decoding acoustic features. **a** Performance decoding vowel identity for one unit, for all time windows (defined by start time and window duration). Black line indicates stimulus onset. **b** Mean performance decoding vowel identity and orthogonal features for all units that were informative about vowel and/or orthogonal feature. Bottom: Difference in performance between vowel and orthogonal feature, illustrating consistent differences in timing of information about vowel and orthogonal features. **c** Decoding performance when reconstructing vowel and orthogonal values from single trial responses of individual units. Markers show best decoding performance of each unit, with unit classification shown as: informative about vowel only (green triangles), orthogonal only (circles), vowel and orthogonal (black diamonds) or neither (gray squares). Single/multi-unit data shown by unfilled/filled markers. Chance performance for vowel, location and voicing was 50% and 20% for F0 and sound level. **d** Cumulative distribution functions (CDFs) showing center times (start time + duration/2) for best performance of each unit when decoding vowel (green) or orthogonal variables (F0: blue; Location: purple; Level: red; Voicing: orange). Units are shown separately by classification as dual feature units (informative about vowel and orthogonal values), or single-feature units (informative about only vowel or orthogonal values). Gray bars represent stimulus duration. Values (*p*) indicate comparison of median center time between decoding of vowel and orthogonal values (dual feature units: sign-rank test; single-feature units: rank-sum test). **e** Number of units informative about vowel/orthogonal values when considering responses across all data. **f** Permutation test comparing the number of units informative about both vowel and orthogonal features observed (black diamonds) vs. chance (unfilled diamonds indicate mean shuffled performance; scatter plots show random performance across 10^4^ iterations after shuffling unit identity). Values (*p*) show the proportion of permutated values above the observed number of units. **g** CDFs for decoding vowel across each orthogonal variable (Vowel) and orthogonal values across vowels (Orth). Data are the same as in **d** but replotted (and recolored for Vowel) by orthogonal dimensions (F0: blue; Location: purple; Level: red; Voicing: orange). Values (*p*) show results from Kruskal–Wallis test

Within the decoding parameter space, we saw stimulus-locked increases in decoding performance (Fig. 2b). For each unit, we tested whether optimized decoding performance was significantly better than that observed when randomly shuffling the decoded feature (permutation test; *p* < 0.05, Supplementary Fig. 5). The proportion of vowel informative units was highest across the dimensions for which animals showed perceptual constancy: Across variation in F0, 37.1% of units (156/421) were informative about vowel identity, 40.7% (55/135) across location and 35.7% (79/221) across level (Fig. 2e). Units informative about vowel identity across one orthogonal feature were also informative across other orthogonal features, suggesting that identity was represented robustly across widely varying acoustic inputs (Supplementary Fig. 7 and Supplementary Table 3). Furthermore, vowel decoding performance did not vary with F0, level or location (Supplementary Fig. 8).

The proportion of vowel informative units was smallest (70/238 units; 29.4%; Supplementary Table 4) for whispered sounds, where animals also failed to generalize behaviorally. Units were also less frequently informative about vowels across voicing and other orthogonal features (Supplementary Fig. 7), and decoding performance differed significantly between voiced and whispered sounds (Supplementary Fig. 8). Thus, impaired behavioral generalization was associated with less widespread and more poorly conserved encoding of vowels.

Perceptual constancy allows humans to extract particular features across changes in sensory input, but we remain sensitive to variation in other dimensions^37^. Consistent with this, we could also decode orthogonal sound features even though they were irrelevant for task performance: 20.4% of units (86/421) were informative about F0 across vowels, 20.4% (45/221) about level, and 30.4% (41/135) about location. Fewer units were informative about F0 or level because more feature values were tested (five) compared to location and voicing (two). If we matched the number of feature values (comparing 149 vs. 459 Hz or 64.5 vs. 82.5 dB SPL), more units were informative about F0 (103/423: 24.4%) and level (44/140: 31.4%). Of all orthogonal features, most units were informative about voicing (46.2%; 110/238), suggesting that whispered stimuli had easily identifiable effects on neural activity. F0 informative units were also more often informative about voicing (Supplementary Fig. 7), suggesting that sensitivity to voicing arises from selectivity for harmonicity. Overall, successful decoding of orthogonal features indicates that, even during vowel identification, auditory cortex maintains sensitivity to multiple sound features.

### Multiplexed and multivariate representations of sound

Sensitivity to multiple stimulus features indicates that, as a population, auditory cortex provides a multivariate representation of sounds. We next asked if individual units provided multivariate representation by testing whether units informative about both vowels and a given orthogonal feature (dual feature units) were more frequently observed than expected from the proportion of units informative about each feature alone. We shuffled unit identity to measure the proportion of dual feature units arising randomly and compared the distribution of shuffled values to the observed occurrence (Fig. 2f). Test permutations were generated using our recorded population, which contained both single and multi-units, and so captured trivial multivariate representations resulting from poor multi-unit isolation. With this control, we observed that significantly more units represented vowel and F0 than expected by chance (permutation test; 10^4^ iterations; *p* = 0.001); similarly, more units were jointly sensitive to vowel and voicing (permutation test, *p* = 0.02). Dual-feature sensitivity was also observed for vowel and location, as well as vowel and level, but their frequency was not significantly greater than chance. Thus vowel, F0 and/or voicing could be represented by the same unit, suggesting that auditory cortex maintains multivariate representations within individual units during perceptual constancy.

Multivariate encoding poses a challenge as changes in firing rate are ambiguous with respect to which stimulus feature is changing. Temporal multiplexing, where neurons represent different stimulus features at distinct time points, may solve this problem^23^. Given we also saw that decoding was time-dependent (Fig. 2a, b); we asked if information about different sound features were systematically represented at different times. To test this, we compared the center time of the decoding window that gave best performance across stimulus features. Timing differences were visualized using cumulative distribution functions (CDFs) across units that were informative about one (single-feature) or multiple stimulus features (dual-feature units)(Fig. 2d).

Information about vowel identity arose significantly earlier than F0, both in dual-feature units that multiplexed F0 and vowel information (time difference (Δ*t*): 153 ms, sign-rank test, *p* = 0.003), and single-feature units representing vowel or F0 (Δ*t*: 233 ms, rank-sum test, *p* = 0.002). Vowel identify was also decoded later than sound location (dual feature units only, Δ*t*: 255 ms, sign-rank, *p* = 0.008) and voicing (single-feature units only, Δ*t*: 245 ms, rank-sum, *p* = 0.016). Vowel identity was decoded earlier than sound level but timing differences were not significant (dual feature units, Δ*t*: 140 ms, sign-rank, *p* = 0.059, single-feature units, Δ*t*: 133 ms, rank-sum, *p* = 0.059). Timing differences were driven by changes in start time of decoding rather than window duration (Supplementary Figs. 9 and 10). Differences in timing for orthogonal variables were also found as F0 and level were decoded significantly later than location and voicing (Supplementary Table 5). Our results thus show temporal multiplexing of sound features in behaving animals, but also that perceptual constancy (for sound level) occurs without significant temporal multiplexing.

We next considered the decoding time of units that were informative about only vowel identity and thus represent only task-relevant and not task-irrelevant sound features. For these units, vowel decoding occurred at similar times for stimulus parameters over which animals successfully generalized (F0, location and level; Fig. 2g). In contrast, decoding of vowel identity across voicing (where animals failed to generalize) was delayed relative to the other conditions. Across all orthogonal features, timing of vowel information differed significantly (Kruskall–Wallis test, *χ*^2^ = 10.41, *p* = 0.015). Post-hoc comparisons (Tukey–Kramer corrected) showed information about vowels across voicing emerged significantly later than vowels varying in F0 (*p* = 0.023) or location (*p* = 0.016), and non-significantly later than vowels varying in level (*p* = 0.155). This was particularly interesting because, despite these units only being informative about vowel identity and not orthogonal features, the timing of vowel identity information was only conserved in the conditions in which the animal could successfully generalize. Such units may thus provide downstream neurons with an invariant and temporally dependent representation of sound identity during successful task performance. When animals failed to generalize vowels across voicing, vowel identity was still encoded within auditory cortex but was significantly delayed in those units providing the most behaviorally relevant representation.

Orthogonal variables were also decoded at different times (Fig. 2g) with significant differences in timing identified for dual (Kruskal–Wallis test, *χ*^2^ = 16.36, *p* = 0.001) and single-feature units (*χ*^2^ = 13.22, *p* = 0.004). Post-hoc comparisons showed F0 was decoded later than location (dual feature, i.e., F0 and location sensitive: *p* = 0.001; single-feature i.e. only significantly F0 or location sensitive: *p* = 0.022). For dual feature units, F0 was also decoded later than voicing (*p* = 0.022), while for single-feature units, sound location was decoded earlier than sound level (*p* = 0.043). Thus, optimizing the temporal parameters of our decoder revealed systematic differences in timing of sound features during perceptual constancy that indicates a time-based, behaviorally relevant structure of auditory encoding.

### Task engagement modulates temporal encoding

**Fig. 3:**
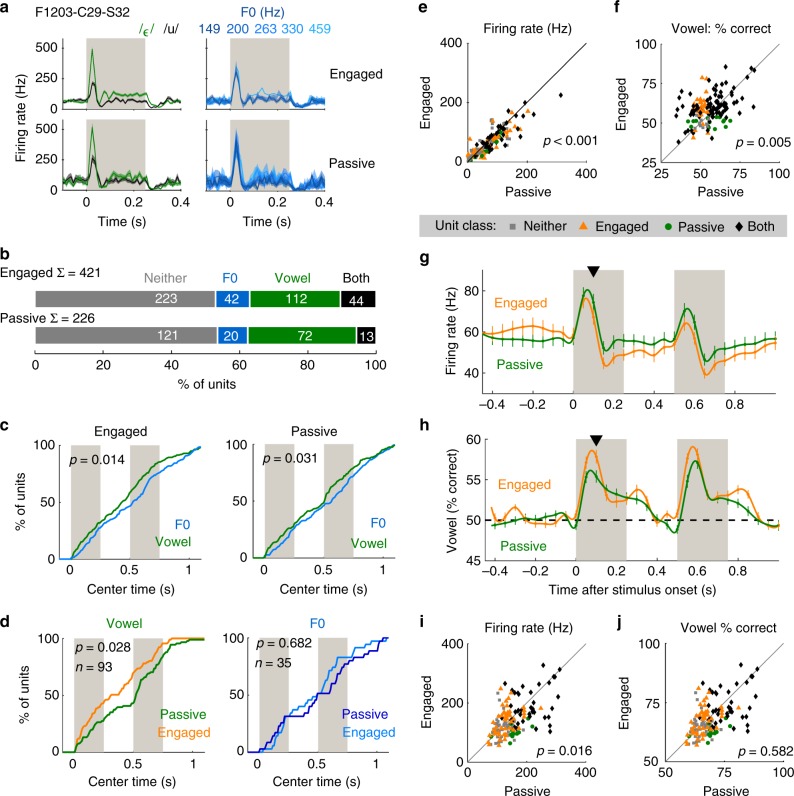
Modulation of auditory processing by task engagement. **a** Example unit responses recorded during task engagement and passive listening to vowels (/u/: black; /ε/: green) varying in F0 (blue). Data are shown as mean ± s.e.m. firing rates across trials (sample sizes given in Supplementary Table 6). **b** Proportion of units informative about vowel identity and/or F0. Σ shows sample size; note that not all units recorded during passive listening were recorded during task engagement. **c** Cumulative density functions (CDF) showing the distribution of decoding time windows giving best performance when decoding vowel (green) and F0 (blue). Data are shown for all units that were informative about vowel and/or F0 in passive or engaged conditions. Values (*p*) indicate comparison of median center time between decoding of vowel and F0 (rank-sum test). **d** CDFs showing center time of windows giving best decoding performance in engaged and passive conditions. Data are shown for units informative about vowel (left, engaged: orange; passive: green) or F0 (right, engaged: light blue; passive: dark blue) during task engagement. Values (*p*) indicate comparison of median center time between engaged and passive conditions (sign-rank test). **e**–**f** Paired comparison of mean firing rate (**e**) and decoding performance (**f**) in the 100 ms after stimulus presentation for units recorded during engaged and passive conditions (*n* = 154). Markers show individual units, labeled by classification as informative about vowel identity in engaged and passive (black diamonds), engaged only (orange triangles), passive only (green circles) or neither condition (gray squares) when decoded using optimized time windows. Values (*p*) indicate comparisons between engaged and passive conditions (sign-rank test). **g**, **h** Paired comparison of firing rate and decoding performance in roving 100 ms windows. Data is shown as mean ± s.e.m. across units (*n* = 154) at 50 ms intervals (spline interpolated across means). Black triangles indicate comparison shown in **e** and **f**. **i**, **j** Firing rate (**i**) and decoding performance (**j**) using optimized time windows (optimized independently in passive and engaged conditions). Values (*p*) indicate comparisons between engaged and passive conditions for all units (sign-rank test). Markers shown as in **e**

To determine if temporal multiplexing depends on task engagement, neural responses to sounds varying in F0 were compared during vowel discrimination and passive listening (Fig. 3a). As during task performance, we could decode vowel identity and F0 from individual units in passive conditions (Fig. 3b) and vowel identity was decoded earlier than F0 in single-feature units (Fig. 3c, Δ*t*: 123 ms, rank-sum test, *p* = 0.014). Temporal multiplexing was, therefore, not restricted to task performance and reflected general auditory processing. However, decoding of vowel identity slowed significantly during passive listening (Fig. 3d, Δ*t*: 110 ms) in units that were vowel informative during task performance (sign-rank test, *p* = 0.028). No effect of task engagement was found on F0 decoding time, or when decoding vowel identity across all units. Thus, timing of information about vowel identity was dependent upon behavioral context, and vowel discrimination was again associated with earlier encoding of vowel identity.

Task engagement suppresses cortical activity^38–40^ and modulates receptive fields of auditory cortical neurons^41–47^. We also observed engagement-related suppression of auditory responses during, but not before, stimulus presentation (Fig. 3e, g): For units recorded across conditions, firing rates in the 100 ms after stimulus onset were significantly lower during engaged than passive conditions (Fig. 3e; sign-rank test, *z* = 3.62, *p* = 2.93 × 10^−4^). In the same period, vowel decoding was significantly better during engaged than passive conditions (Fig. 3f, sign-rank test, *z* = −2.83, *p* = 0.005). Engagement-related enhancement of decoding performance was limited to sound onset and offsets (Fig. 3h), while changes in decoding performance and firing rate were not correlated (linear regression, *p* > 0.05).

As decoding performance using fixed time windows underestimated information content (Supplementary Fig. 6), we also compared spike rates and vowel decoding in the window giving best decoding performance, optimized for passive and engaged conditions independently. Firing rates in optimized windows were lower in engaged than passive conditions (Fig. 3i; all units: sign-rank test, *z* = 3.20, *p* = 0.001, vowel informative units: *z* = 2.41, *p* = 0.016), but engagement did not improve decoding performance: For units informative about vowel identity during the task, decoding performance was statistically indistinguishable (Fig. 3j; sign-rank test, *z* = −0.55, *p* = 0.582), while across all units, a small but significant decline in decoding performance was observed (sign-rank test, *z* = 2.15, *p* = 0.032). Thus, the main effect of task engagement was to change the time at which vowel information was decoded rather than the amount of information available. Altogether, these results indicate further that perceptual constancy relies on reliable timing of information about vowel identity.

### Training does not enhance representation of vowel sounds

Perceptual learning enhances neural discrimination of sound features such as level, frequency^9,48^, and timbre^49^. However, it’s unclear whether perceptual learning is required for ferrets to accurately discriminate vowels, as neurons in anesthetized naive ferrets are already sensitive to vowel identity^25^. To test whether behavioral training affects representations of vowels varying in F0, we compared neuronal responses in passive listening when (1) trained animals were presented with trained and untrained stimuli, or (2) the same vowels were presented to trained and naive animals (Supplementary Fig. 11).

Consistent with anesthetized data, units in untrained animals discriminated vowels well, as did units in trained animals presented with untrained vowels. We found that training was associated with a degraded representation of vowel identity, reflected by small but significant reductions in decoding performance (trained vs. untrained sounds, sign-rank test, *p* = 0.016; trained vs. untrained subjects, rank-sum test, *p* = 0.003). Thus, training did not enhance the representation of vowel sounds, suggesting that naive ferrets may naturally distinguish vowel timbre. This is consistent with the role of timbre in the ferret’s own vocalizations^33^ and rapid behavioral generalization of vowel identity to novel sounds (Fig. 1d). Thus, training most likely conditioned animals to associate existing auditory representations with behavioral responses and liberate cortical resources for representing non-sensory features related to behavior.

### From sound to behavior

Behavioral training was necessary to measure animals’ perception of sounds across variations in acoustic input. Our previous analyses used only trials that animals performed correctly, as these provide the clearest insight into auditory processing. However on correct trials, sound identity is confounded with behavioral response as each vowel is associated with a specific choice to respond left or right. Neurons may represent choice as well as sound identity^50,51^ and so we investigated how behavior affected neural processing by comparing activity on correct and error trials.

We first asked if representations of sound features were dependent on the animal’s behavior by decoding neural responses on error trials, in which the same range of stimuli were presented, yet subjects made opposite responses to correct trials. If unit activity was purely stimulus-driven, then decoding should be similar regardless of trial accuracy; whereas significant differences in decoding would reveal a relationship between auditory cortex and behavior. Decoding performance for vowel identity and orthogonal features was indeed significantly worse on error trials than correct trials (Wilcoxon sign-rank: *p* < 0.001, Supplementary Fig. 12 and Supplementary Tables 7 and 8), suggesting that cortical activity and behavior were linked.

**Fig. 4:**
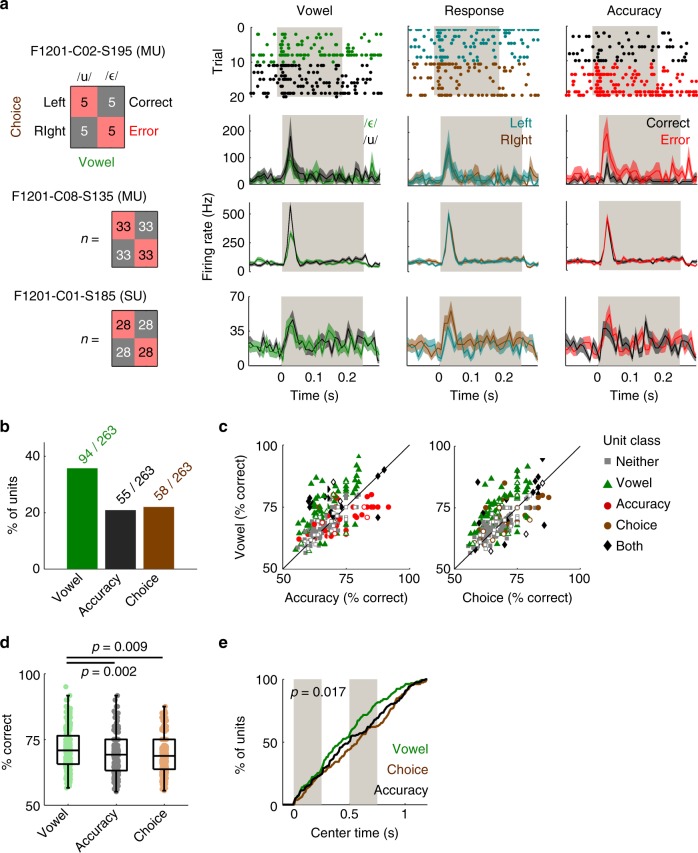
Auditory cortical neurons encode sound, choice and accuracy. **a** Analysis design for matching equal numbers of responses to each vowel (/u/: black; /ε/: green), behavioral choice (left: cyan; right: brown) and trial outcome (correct: black; error: red). Data shown as raster plots of spike times on each trial for one unit and PSTHs representing mean ± s.e.m. firing rate across trials for three units (two multi-units [MU] and one single unit [SU]). Gray bars show the first stimulus token. Trial contingency (i.e., respond left for /ε/) shown as an example on which one ferret was trained (F1217). **b** Percentage of units informative about sound, choice and/or accuracy in matched data. **c** Performance decoding sound, choice, and accuracy across all units. Markers show individual units labeled by classification as informative about vowel only (green triangles), accuracy only (red circles), choice only (brown circles), no variable (neither: gray squares) or multiple variables (both: black diamonds). The terms neither and both refer to the dimensions shown within each scatter plot (e.g., about both vowel and choice) rather than across plots. Single/multi-unit data shown by unfilled/filled markers. **d** Comparison of performance decoding sound, choice and accuracy; boxplots show median, interquartile range (box) and 99.3% intervals (whiskers). Values (*p*) show significant Tukey–Kramer corrected pairwise comparisons following Kruskal–Wallis test. **e** Cumulative distributions showing center times for best performance when decoding vowel (green), choice (brown) and accuracy (black). Gray bars represent the duration of the each token within the stimulus. Data shown for all units that were informative about one or more variables. Value (*p*) shows significant difference in time of decoding identity, choice and accuracy (Kruskal–Wallis test)

Behavioral errors may be driven by impaired cortical representation of sounds, or cortical responses may convey choice signals for the animal’s response. We observed that choice-related decoding declined more markedly on error trials than stimulus decoding (Supplementary Fig. 12) suggesting that cortical representations of stimulus identity are less substantially impaired when animals made mistakes. However, this analysis was limited because animals made fewer errors than correct responses. To advance further, we subsampled datasets with equal numbers of correct and error trials, and matched sample sizes of vowels and behavioral responses (Fig. 4a) to independently contrast decoding of sound, choice and task accuracy. Our matched datasets brought together data in which vowels varied across F0, location and level, but excluded sounds below 60 dB SPL or whispered vowels that animals failed to generalize across. We additionally excluded trials with behavioral responses within 1 s of second sound onset, to avoid confounds related to inclusion of trial outcome within the decoding window.

Information about sound identity was more widespread than about behavioral variables: 35.7% of units (94/263, permutation test, *p* < 0.05) were significantly informative about sound identity, 22.1% (58/263) about choice and 20.9% (55/263) about accuracy (Fig. 4b). Decoding was also better for vowel identity (mean ± s.e.m. performance: 71.5 ± 0.47%) than for accuracy (69.2 ± 0.45%) or choice (69.4 ± 0.43%) (Fig. 4c). Decoding performance differed significantly across all variables (Kruskal–Wallis test: *χ*^2^ = 13.6, *p* = 0.001) with pairwise comparisons (Fig. 4d) confirming that vowel decoding was better than choice (Tukey–Kramer corrected, *p* = 0.009) and accuracy (*p* = 0.002). There was no significant difference between choice and accuracy. Overall, the animal’s behavioral choice and accuracy could thus be decoded from unit activity, but auditory cortex predominantly represented sound identity.

Identity, choice, and accuracy were also decoded at different times: Information about sound identity emerged earliest, followed by accuracy and then choice (Fig. 4e). For 158 units that were informative about sound identity, choice and/or accuracy, the time of best decoding differed significantly between dimensions (Kruskal–Wallis test, *χ*^2^ = 8.13, *p* = 0.017) with choice represented later than sound identity (Post-hoc pairwise comparison, Tukey–Kramer corrected, Δ*t* = 100 ms, *p* = 0.013). Timing of accuracy information was not significantly different from sound or choice (*p* > 0.1). Thus temporal multiplexing occurred for behavioral, as well as sensory variables, in a sequence consistent with sensory-motor transformation.

### Population decoding accuracy exceeds behavioral performance

Our analysis of matched datasets contained equal proportions of errors and correct trials and so animals’ behavioral performance over these trials would correspond to 50%. Despite this, we could decode vowel identity from the activity of many units with better accuracy. This raises the question of how neural encoding of sounds compared to behavioral discrimination. To answer this, we used a population decoding approach to approximate how downstream neurons within the brain might read-out activity from auditory cortex.

**Fig. 5:**
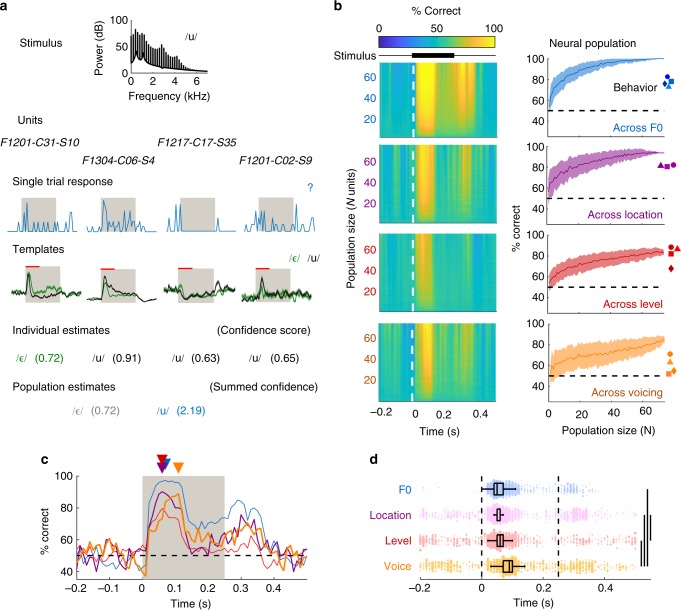
Population decoding can match behavioral performance. **a** Schematic illustrating population decoder using four constituent units: On each trial, vowel identity (top row) was estimated independently by each unit within the population (second row) using the distance between the pattern of neural activity on that trial (third row) and templates built from responses on all other trials (fourth row). For each unit, we obtained an estimate of the stimulus feature and a weight (individual estimate and confidence score; fifth row). Confidence weights for each vowel were summed (sixth row) to give a population estimate of each vowel’s likelihood, with the maximum weight giving the population estimate. Red lines above templates indicate the time window of response considered, which was consistent across units and roved in the main analysis. The decoding procedure was repeated across trials using leave-one-out cross-validation to estimate population performance. Units contributing to each population were randomly subsampled. **b** Performance decoding vowel identity across F0, location, level or voicing using varying populations size and neural activity measured in roving 100 ms windows. Surface plots (left) show mean performance of populations (*n *= 100) sampled with different constituent units. Line plots (right) show decoding performance as a function of population size using neural activity between 0 and 100 ms after stimulus onset. Error bars show standard deviation across populations (*n* = 100) with different constituents. Data points show the behavioral performance of each ferret (F1201: circles; F1203: triangles; F1217: squares; F1304: diamonds) for each orthogonal dimension. **c** Mean decoding performance across all population sizes reveals later decoding of vowels across voicing than other features (F0: blue; Location: purple; Level: red; Voicing: orange). Triangle markers indicate the time of peak performance for each orthogonal condition. **d** Comparison of best decoding performance across every population tested. Marker size indicates population size (1–74 units) with marker position showing the time at which each population decoded vowels best. Boxplots show median, interquartile range (box) and 99.3% intervals (whiskers). Lines show significant comparisons across dimensions (permutation test, *p* < 0.001)

Population decoders summed estimates of vowel identity from a variable number of units, weighted by the relative spike-distance between decoding templates and test response (Fig. 5a; see Methods). Estimates were made using responses sampled in roving 100 ms windows, with decoding performance peaking at stimulus onset and offset (Fig. 5b). Across both correct and error trials, vowel identity could be decoded with 100% accuracy when sounds varied in F0, even though animals’ performance never exceeded 90% correct. Similarly, we decoded vowels across sound location with > 93% and voicing with > 80% performance when animals’ performance was below 85% and 72% respectively. Decoding of vowel across sound level was similar when decoding neural populations or during behavior. Thus information available in auditory cortex was sufficient to discriminate vowels better than animals actually did.

We also analyzed timing of information, focussing on the time decoding performance peaked. Decoding of vowels across voicing was slower than decoding of vowel across F0, location or level (Fig. 5c). For all populations tested, we asked when each population performed best in time (Fig. 5d) and compared the distribution of timing values between dimensions. Decoding vowels across voicing peaked significantly later than across F0, level or location (permutation test, *p* < 0.001). Performance decoding vowels across location also peaked significantly earlier than across level, but later than across F0 (*p* < 0.05). These timing differences in population decoding were consistent with results from individual units, suggesting that information timing may be as critical to discriminating sounds as information content: Decoding of vowels was slowest across whispered conditions, when animals failed to generalize vowels, even though both individual unit and population decoders performed well.

## Discussion

Here, we demonstrate that auditory cortical neurons represent vowel identity reliably across orthogonal acoustic transformations that mirror those preserved in perceptual constancy. The neural representation provided by many neurons was multivariate, as units represented multiple stimulus features, and temporally multiplexed, as variables were best represented at different times. Multivariate encoding extended to behavioral dimensions as units represented subjects’ choice and accuracy, and decoding performance differed between correct and error trials. Altogether our findings demonstrate that auditory cortex provides sufficient tolerance across variation in sensory input and behavior to accurately represent the identity of target sounds during perceptual constancy.

Ferrets identified vowels by their spectral timbre while sounds varied across major acoustic dimensions that are key to real-world hearing, including F0 (that determines voice pitch), sound location and level. Both animals and neurons generalized across similar acoustic dimensions (F0, space etc.), while neurons represented vowel identity and F0 during task performance and when passively listening (which did not require training). Encoding of multiple features of speech-like sounds, sometimes by the same units, supports previous findings of both distributed coding and temporal multiplexing of multiple stimulus features in auditory cortex^25,52,53^. It is notable that these earlier studies were performed in anesthetized ferrets and reached very similar conclusions for vowels varying in F0 and virtual acoustic space^25^ suggesting that general principles of auditory processing are observable across anesthetized and behavioral states. However, because we tested neural representations of phonemes in behaving animals, we could also show that orthogonal variables (e.g., F0) were encoded, even when potentially disruptive to behavior. This is consistent with our ability to perceive multiple dimensions of sounds, but raises questions about how behaviorally relevant sound features are extracted by downstream neurons; specifically, where in the brain does multivariate encoding give way to univariate representation of only task-relevant dimensions?

One possibility is that univariate encoding already exists within auditory cortex, in the responses of units that were informative about vowel identity but not orthogonal features. Although such units are not the only class recorded, such neurons could provide a selective, task-relevant output for downstream neurons to identify sounds robustly. The connectivity and causal relevance of vowel informative units remains to be tested, although interactions are likely with areas such as prefrontal and higher-order auditory cortex (dPEG) showing selectivity for behaviorally relevant sounds^41,54–57^. Correspondingly, units in such areas would be expected to filter out sensitivity to orthogonal sound features so that task-irrelevant information is lost.

We decoded vowel identity and orthogonal variables independently and with minimal selection of neural response time windows. This approach showed that vowel identity and orthogonal features were best decoded in distinct time windows. Temporal multiplexing by units mirrored the time-course of sound perception: Decoding of vowel identity and sound location earlier than voicing or F0 is consistent with perception of sound location and vowel identity at sound onset^58,59^, while listeners require longer to estimate F0^23,60,61^ and sound level^62,63^.

Our results, together with previous work^23^, demonstrate that multiplexing is relevant across cortical states (anesthetized, awake, attentive) and a general feature of auditory processing. In contrast, encoding of vowel identity early after stimulus onset was specific to conditions when animals discriminated vowels: When animals failed to generalize vowels across voicing, or listened passively, we observed a significant delay in decoding vowel identity—both with individual units and neural populations. For whispered sounds, slower encoding may be explained in part by the noisy sampling of the spectral envelope when compared to harmonic sounds; however acoustic differences cannot explain changes in encoding of the same sounds in passive and engaged conditions. Instead our results suggest that timing of vowel encoding is dynamic and depends on both stimulus properties and behavioral state. Moreover, behavioral discrimination only occurred when vowels were represented in a specific time window, indicating that downstream regions responsible for decision making may sample auditory cortical information in critical time windows. This theory would predict that temporally selective lesions of auditory cortical activity at stimulus onset (but not during sustained periods of sounds) should disrupt task performance—though this remains to be tested in behaving ferrets.

We also demonstrated that auditory cortex represents behavioral variables: Many units encoded information about the animals’ choice and/or accuracy, and decoding of sound features was impaired on error trials. Such findings are consistent with previous reports of choice-related activity^39,46,51^; however we also recorded units that were sensitive to accuracy and thus predictive of upcoming mistakes. When combined with results from population decoding, in which cortical activity could identify vowels better than animals’ behavior, we must ask: why do animals make mistakes?

It’s possible that errors arise from inattention, which has a distinct neural signature^64^ that our decoder uses to distinguish correct and error trials. At present it is unclear whether the accuracy signal we decode reflects such attentional lapses, or arises from interactions between representations of sound identity and behavioral choice, a representation of confidence in auditory processing, or anticipation of reward^65^. Future experiments systematically manipulating confidence or reward value may explain the precise nature of accuracy information reported here.

Overall, our results show that tolerant representations of vowel identity exist when animals show perceptual constancy. We found that principles of auditory processing such as multivariate representations of sound features and temporal multiplexing occur during perceptual constancy, and do not require training or task engagement. However representation of sound identity early after stimulus onset was associated with successful sound discrimination, suggesting that timing of acoustic representations is essential for auditory decision making. Animals failed to use all the information available in the populations of auditory cortical units, indicating that animals’ final behavioral responses are governed by factors including, but also extending beyond, auditory cortex.

## Methods

### Animals

Subjects were four pigmented female ferrets (1- to 5-years-old) trained to discriminate vowels across fundamental frequency, sound level, voicing, and location^15,17^. Each ferret was chronically implanted with Warp-16 microdrives (Neuralynx, MT) housing 16 independently moveable tungsten microelectrodes (FHC, Bowdoin, ME) positioned over primary and posterior fields of left and right auditory cortex. A further four ferrets (also pigmented females, 1- to 3-years-old) implanted with the same microdrives were used as naive animals for passive recording. These animals were trained in either a two-alternative relative sound localization or go/no-go multisyllabic word identification task that did not involve the synthetic vowel sounds presented here.

Subjects were water restricted prior to testing; on each day of testing, subjects received a minimum of 60 ml kg^-1^ of water either during testing or supplemented as a wet mash made from water and ground high-protein pellets. Subjects were tested in morning and afternoon sessions on each day for up to 5 days in a week. Test sessions lasted between 10 and 50 min and ended when the animal lost interest in performing the task.

The weight and water consumption of all animals was measured throughout the experiment. Regular otoscopic examinations were made to ensure the cleanliness and health of ferrets’ ears. Animals were maintained in groups of two or more ferrets in enriched housing conditions. All experimental procedures were approved by local ethical review committees (Animal Welfare and Ethical Review Board) at University College London and The Royal Veterinary College, University of London and performed under license from the UK Home Office (Project License 70/7267) and in accordance with the Animals (Scientific Procedures) Act 1986.

### Microdrive implantation

Microdrives were surgically implanted in the anesthetized ferret under sterile conditions. General anesthesia was induced by a single intramuscular injection of medetomidine (Domitor; 0.1 mg kg^-1^; Orion, Finland) and ketamine (Ketaset; 5 mg kg^-1^; Fort Dodge Animal Health, Kent, UK). Animals were intubated and ventilated, and anesthesia was then maintained with 1.5% isoflurane in oxygen throughout the surgery. An i.v. line was inserted and animals were provided with surgical saline (9 mg kg^-1^) intravenously throughout the procedure. Vital signs (body temperature, end-tidal CO2 and the electrocardiogram) were monitored throughout surgery. General anesthesia was supplemented with local analgesic (Marcaine, 2 mg kg^-1^, Astra Zeneca) injected at the point of midline incision. Under anesthesia, the temporal muscle overlying the skull was retracted and a craniotomy was made over the ectosylvian gyrus. Microdrives were then placed on the surface of the brain and embedded within silicone elastomer (Kwik-Sil, World Precision Instruments) around the craniotomy, and dental cement (Palacos R + G, Heraeus) on the subject’s head. Ground and reference signals were installed by electrically connecting the microdrive to bone screws (stainless steel, 19010-100, Interfocus) placed along the midline and rear of the skull (two per hemisphere). A second function of the bone screws was to anchor bone cement to the skull; this was also facilitated by cleaning the skull with citric acid (0.1 g in 10 ml distilled water) and application of dental adhesive (Supra-Bond C&B, Sun Medical). Some temporal muscle and skin were then removed in order to close the remaining muscle and skin smoothly around the edges of the implant. Animals were allowed to recover for a week before the electrodes were advanced into auditory cortex. Pre-operative, peri-operative and post-operative analgesia and anti-inflammatory drugs were provided to animals under veterinary advice.

### Confirmation of electrode position

At the end of the experiment, animals were anesthetized with medotomidine (0.05 mg kg^-1^, Orion) and ketamine (2.5 mg kg^-1^, Vetoquinol, UK) followed by overdose with intraperitoneal administration of pentobarbitone (300 mg kg^-1^_,_ Pentoject, Animal Care). Animals were then transcardially perfused with 0.9% Saline, followed by 4% paraformaldehyde in phosphate buffered solution. The brain was then removed and stored in paraformaldehyde for ≥ 1 week before cryoprotection in sucrose (30% in dH_2_O), freezing in dry ice and histological sectioning (cryostat, section thickness: 50μm). Prior to sectioning, the brain was photographed to record the position of electrode penetrations on the cortical surface. Sections were then mounted in 3% gelatin on microscope slides and Nissl stained to visualize the tissue. Electrode tracks were visible as local disruption of tissue, and the pattern of tracks through the tissue was aligned with that observed across the cortical surface of the intact brain. Electrodes that did not enter the Ectosylvian Gyrus were removed from the dataset. We also discarded data from electrodes recorded below the cortical laminae.

### Apparatus

Ferrets were trained to discriminate sounds in a customized pet cage (80 × 48 × 60 cm, length × width × height) within a sound-attenuating chamber (IAC) lined with sound-attenuating foam. The floor of the cage was made from plastic, with an additional plastic skirting into which three spouts (center, left and right) were inserted. Each spout contained an infra-red sensor (OB710, TT electronics, UK) that detected nose-pokes and an open-ended tube through which water could be delivered.

Sound stimuli were presented through two loud speakers (Visaton FRS 8) positioned on the left and right sides of the head at equal distance and approximate head height. These speakers produce a smooth response ( ± 2 dB) from 200 Hz to 20 kHz, with an uncorrected 20 dB drop-off from 200 to 20 Hz when measured in an anechoic environment using a microphone positioned at a height and distance equivalent to that of the ferrets in the testing chamber. A light-emitting diode (LED) was also mounted above the center spout and flashed (flash rate: 3 Hz) to indicate the availability of a trial. The LED was continually illuminated whenever the animal successfully made contact with the IR sensor within the center spout until a trial was initiated. The LED remained inactive during the trial to indicate the expectation of a peripheral response and was also inactive during a time-out following an incorrect response.

The behavioral task, data acquisition, and stimulus generation were all automated using custom software running on personal computers, which communicated with real-time signal processors (RZ2 and RZ6, Tucker-Davis Technologies, Alachua, FL).

### Task design

Ferrets discriminated vowel identity in a two-choice task^15^. On each trial, the animal was required to approach the center spout and hold head position for a variable period (0–500 ms) before stimulus presentation. Each stimulus consisted of a 250 ms artificial vowel sound repeated once with an interval of 250 ms. The vowel sound was repeated here to maintain the same task design as previous studies^15,17^, although subsequent testing demonstrated that repetition was not necessary for successful task performance (Supplementary Fig. 1). Animals were required to maintain contact with the center spout until the end of the interval between repeats (i.e., 500–1000 ms after initial nose-poke) and could then respond at either left or right spout. Correct responses were rewarded with water delivery whereas incorrect responses led to a variable length time-out (3 to 8 s). To prevent animals from developing biases, incorrect responses were also followed by a correction trial on which animals were presented with the same stimuli. Correction trials and trials on which the animal failed to respond within the trial window (60 s) were not analyzed. The only exception to this protocol was for whispered sounds, which we presented as probe sounds in 10–20% of trials, on which any response was rewarded and correction trials did not follow.

Animals were initially trained to discriminate vowels that were constant in F0, voicing location and level, at which point sounds were then roved in level over a 6 to 12 dB range. Following this, animals were exposed to vowels varying in F0 with two different F0s being tested (149 and 200 Hz). We then progressively extended the range of F0s used in testing by including higher F0s. We later increased the range of sound levels over which animals were tested from 12 up to 30 dB SPL, and introduced variation in voicing and sound location. Features (F0, level etc.) were trained and tested separately on different sessions but the order of sessions varied pseudo-randomly within days and weeks such that there was no systematic progression from one feature to another. Neural data was only recorded once the animals were fully trained and performance had plateaued.

We recorded neural activity during task performance, and also under passive listening conditions, in which animals were provided with water at the center port to recreate the head position and motivational context occurring during task performance. Sounds were presented with the same two-token stimulus structure as during task performance, with a minimum of 1 s between stimuli. During test sessions, sound presentation began once the animal approached the center spout and began licking and ended when the animal became sated and lost interest in remaining at the spout.

### Stimuli and behavioral testing

Stimuli were artificial vowel sounds synthesized in MATLAB (MathWorks, USA) based on an algorithm adapted from Malcolm Slaney’s Auditory Toolbox (https://engineering.purdue.edu/~malcolm/interval/1998-010/). The adapted algorithm simulates vowels by passing a sound source (either a click train to mimic a glottal pulse train for voiced stimuli, or broadband noise for whispered stimuli) through a biquad filter with appropriate numerators such that formants are introduced in parallel. Four formants (F1-4) were modeled: three subjects were trained to discriminate /u/ (F1-4: 460, 1105, 2857, 4205 Hz) from /ε/ (730, 2058, 2857, 4205 Hz) while one subject was trained to discriminate /a/ (936, 1551, 2975, 4263 Hz) from /i/ (437, 2761, 2975, 4263 Hz). Selection of formant frequencies was based on previously published data^15,28^ and synthesis produced sounds consistent with the intended phonetic identity. Formant bandwidths were kept constant at 80, 70, 160, and 300 Hz (F1-4 respectively) and all sounds were ramped on and off with 5 ms cosine ramps.

To test perceptual constancy, we varied the rate of the pulse train to generate different fundamental frequencies (149, 200, 263, 330, and 459 Hz) and used broadband noise rather than pulse trains to generate whispered vowels. For sound level, we simply attenuated signals in software prior to stimulus generation. For sound location, we presented vowels only from the left or right speaker whereas all other tests sounds were presented from both speakers. Across variations in F0, voicing and space, we fixed sound level at 70 dB SPL. For tests across sound level and location, voiced vowels were generated with 200 Hz fundamental frequency. In tests of neural encoding in passively listening animals (both trained and untrained), we presented vowels /u/ and /ε/ at 70 dB SPL with the same F0s (149, 200, 263, 330, and 459 Hz) that task-engaged animals discriminated. Sound levels were calibrated using a Brüel & Kjær (Norcross, USA) sound level meter and free-field ½ inch microphone (4191) placed at the position of the animal’s head during trial initiation.

### Neural recording

Neural activity in auditory cortex was recorded continuously throughout task performance. On each electrode, voltage traces were recorded using System III hardware and OpenEx software (Tucker-Davis Technologies, Alachua, FL) with a sample rate of 50 kHz. For extraction of action potentials, data were bandpass filtered between 300 and 5000 Hz and motion artefacts were removed using a decorrelation procedure applied to all voltage traces recorded from the same microdrive in a given session^66^. For each channel within the array, we identified spikes (putative action potentials) as those with amplitudes between -2.5 and -6 times the root-mean squared value of the voltage trace and defined waveforms of events using a 32-sample window centered on threshold crossings.

For data obtained in task-engaged animals, waveforms were then interpolated (128 points) and candidate events combined across sessions within a test run for spike sorting. Waveforms were manually sorted using MClust (A.D. Redish, University of Minnesota, http://redishlab.neuroscience.umn.edu/MClust/) so that candidate events were assigned to either single-unit, multi-unit clusters or residual hash clusters. Single units were defined as those with less than 1% of inter-spike intervals shorter than 1 millisecond.

We identified 502 sound-responsive units (141 single units; 28.1%) in task-engaged animals as those whose stimulus evoked response within the 300 ms after onset of first token differed significantly from spontaneous activity in the 300 ms before making contact with the spout (Sign-rank test, *p* < 0.05). In passive conditions, we identified responsive units using a similar comparison; but using spontaneous activity measured in the 300 ms before stimulus presentation. In comparisons of neural data between task-engaged and passive animals, we only used multi-unit activity obtained prior to spike sorting.

### Decoding procedure

We decoded stimulus features (e.g., vowel identity, F0 etc.) on single trials using a simple spike-distance decoder with leave-one-out cross-validation (LOCV). For every trial over which an individual unit was tested in a given dataset (e.g., vowels varied across F0 during task performance), we calculated template responses for each stimulus class (e.g., each vowel or each F0) as the mean peri-stimulus time histogram (PSTH) of responses on all other trials. We then estimated the stimulus feature on the test trial as the template with the smallest Euclidean distance to the test trial (Supplementary Fig. 3). Where equal distances were observed between test trial and multiple templates, we randomly estimated (i.e., guessed) which of the equidistant templates was the true stimulus feature. This procedure was repeated for all trials and decoding performance was measured as the percentage of trials on which the stimulus feature was correctly recovered. Although this approach was simple and did not account for the variance of neural activity, it provided an intuitive relationship between neural activity and information content that we could use with small datasets (sample sizes down to five trials per condition). Robustness to sample size was particularly important because the animal’s behavior determined the number of trials in each condition and we aimed to analyze as many units as possible rather than develop a more sophisticated decoder.

Auditory cortical units showed a wide variety of response profiles that made it difficult to select a single fixed time window over which to decode neural activity. To accommodate the heterogeneity of auditory cortical neurons and identify the time at which stimulus information arose, we repeated our decoding procedure using different time windows (*n* = 1550) varying in start time (-0.5 to 1 s after stimulus onset, varied at 0.1 s intervals) and duration (10 to 500 ms, 10 ms intervals) (Fig. 2a, b and Supplementary Fig. 4). Within this parameter space, we then reported the parameters that gave best decoding performance, and where several parameters gave best performance, we reported the time window with earliest start time and shortest duration.

To assess the significance of decoding performance, we conducted a permutation test in which the decoding procedure (including temporal optimization) was repeated 100 times but with the decoded feature randomly shuffled between trials to give a null distribution of decoder performance (Supplementary Fig. 5). The null distribution of shuffled decoding performance was then parameterized by fitting a Gaussian probability density function, which we then used to calculate the probability of observing the real decoding performance. Units were identified as informative when the probability of observing the real performance after shuffling was <0.05. Parameterization of the null distribution was used to reduce the number of shuffled iterations over which decoding was repeated. This was necessary because the optimization search for best timing parameters dramatically increased the computational demands of decoding.

### Population decoding

To decode vowel identity from the single trial responses of populations of units, we simply the summed the number of units that estimated each stimulus, weighted by the confidence of each unit’s estimate, and took the stimulus with the maximum value as the population estimate on that trial (Fig. 4a). Confidence weights for individual unit (*w*) estimates were calculated as:

1 $$w = 1 - \frac{{d_{{\mathrm{min}}}}}{{\mathop {\sum }\nolimits_{j = 1}^n d_j}}$$

Where *n* was the number of stimulus classes (e.g., vowel identities) and *d* was the spike-distance between the test trial response and response templates generated for each stimulus class. Here, *d*_min_ represents the minimum spike-distance that corresponded to the estimated stimulus for that unit.

We tested populations of up to 74 units, by which point decoder performance had typically saturated. The decision to use this maximum population size was motivated by (1) the minimum number of units that were informative about sound features, and (2) the number of trials each unit was tested with. We only included units for which we recorded neural responses on at least 8 trials for each vowel. Both correct and error trials were included in decoding. Populations were constructed first by selecting the units that perform best at decoding vowel identity on correct trials at the individual unit level (Fig. 2c). Within this subpopulation, we randomly sampled 100 combinations of units without replacement from the large number of possible combinations of units available.

### Data analysis

Unless otherwise stated (e.g., permutation tests), all statistical tests were two-tailed.

Behavior (uniformity of performance): Perceptual constancy was reported when the orthogonal factor (e.g., F0) did not significantly affect task performance, i.e. the likelihood of responding correctly. To test this, we analyzed the proportion of correct trials as a function of each orthogonal dimension using a logistic regression (Supplementary Table 2). Regressions were performed separately for each animal, and each orthogonal dimension, and any significant effect (*p* < 0.05) was reported as a failure of constancy. We also asked if an animal’s performance at specific orthogonal values was better than chance (50%) using a binomial test (*p* < 0.001, Supplementary Table 1).

Behavior (generalization): To test if animals generalized vowel identity across orthogonal values (e.g., F0) we compared performance with stimulus experience. Subjects were initially trained on a specific orthogonal value (e.g., F0 = 200 Hz) and then exposed to varying orthogonal values (e.g., F0 = 149, 263, 330, and 459 Hz). Each ferret’s performance was computed in windows beginning with the first trial experienced, and extending out to consider progressively longer durations. Initial performance was compared with chance by randomizing the required response across trials and recalculating percent correct for each time window. To find the number of trials at which animals first discriminated vowel identity with novel sounds, we used a permutation test to measure chance performance on 10^4^ iterations and identified significant performance as that with a false-positive probability of below 0.001. The values reported in Fig. 1e show the minimum number of trials at which performance was significant. We also compared initial performance to long-term accuracy to illustrate the relevance of generalization to behavior across the study (Supplementary Fig. 2). To measure long-term performance, we randomly selected sequences of trials taken from the entire dataset (i.e., not those trials the animal first experienced the particular stimulus) with a set window length and recalculated performance. This procedure was repeated 10^4^ times. For analysis of generalization across F0, we also included two additional F0s (409 and 499 Hz) for which data was only collected prior to electrode implantation and thus not included in the main text.

Neural activity: The times of spikes was referenced to the onset of the stimulus on each trial and used to create raster and peri-stimulus time histograms. In our analysis of task engagement and training, we measured on each trial the firing rate in 100 ms bins after stimulus onset at 50 ms intervals. For paired comparisons, firing rates in engaged and passively listening animals were compared using a Wilcoxon sign-rank test. For unpaired analyses, we normalized firing rates in these bins relative to the firing rate in a pre-stimulus baseline period in the 450 ms before stimulus onset (passively listening animals) or before the animal began waiting at the center spout (task-engaged animals). Across passively listening groups presented with familiar/unfamiliar sounds (Supplementary Fig. 11), we compared normalized firing rates and baseline firing rates (i.e., the normalization factors in each condition) across groups using a Kruskal–Wallis test with pairwise post-hoc comparisons performed with Tukey–Kramer correction for multiple comparisons.

Individual unit decoding: In addition to classifying whether units were informative about a particular stimulus feature (permutation test, *p* *<* 0.05), we also compared decoding performances (Figs. 2c, 3f, j, 4c, d, Supplementary Fig. 6, 11b–g, and 12e). When comparing decoding performance across more than two conditions (i.e., when decoding vowel, accuracy or choice; Fig. 4d), data were analyzed using a Kruskal–Wallis test with Tukey–Kramer corrected post-hoc comparisons where relevant. When comparing two conditions directly, we used a Wilcoxon sign-rank test for paired data (e.g., comparing performance on correct and error trials; Supplementary Fig. 11b). For comparison of changes in decoding performance between conditions (e.g., decoding sound identity in naive and trained animals; Supplementary Fig. 11e), we used a Wilcoxon rank-sum comparison for unpaired data.

Timing: For each unit, we determined the time window after stimulus onset for which we achieved best decoding performance (Supplementary Fig. 4) and took the window center (Fig. 2d), start time (Supplementary Fig. 9) or window duration (Supplementary Fig. 10). We then compared the change in parameter value (e.g., change in center time) for best decoding of vowel identity and orthogonal dimensions using a Wilcoxon rank-sum test. The same approach was used when comparing the timing of decoding vowel identity and F0 in task-engaged and passively listening animals (Fig. 3c, d). We also compared the times of best decoding of vowel identity across orthogonal dimensions using a Kruskal–Wallis test with Tukey–Kramer correction for post-hoc comparisons (Fig. 2g). We used the same approach to compare the decoding of orthogonal dimensions, and decoding of vowel identity, behavioral choice and accuracy (Fig. 4e). Time differences (Δ*t*) reported in the main text were shown as the median difference in center time using a paired comparison (dual feature units) or the difference in median center time using an unpaired comparison (single-feature units).

Datasets matched for vowel, choice and accuracy (Fig. 4): To study the tolerance of a given unit to behavioral as well as acoustic variables, we subsampled neural responses from all conditions in which animals showed perceptual constancy: Specifically we included sounds varied across F0, sound location and sound level above 60 (three ferrets) or 70 dB SPL (one ferret). We excluded all data when sounds were whispered. To prevent trial outcome (water reward or time-out) from confounding accuracy signals, we also excluded trials on which animals responded within one second of stimulus onset. Following pooling and exclusion, we balanced datasets for the number of each vowel, choice and trial outcome by randomly selecting *N* trials, where *N* was the minimum number of trials in which any one condition (e.g., left responses to /u/) was tested. As with our earlier decoding analysis, we only considered units for which *N* ≥ 5. We then decoded vowel identity, behavioral choice and accuracy using the same LOCV decoding procedure described above. We compared decoding performance for vowel identity, choice and accuracy across all units with a Kruskal–Wallis anova and post-hoc comparisons using the Tukey–Kramer correction (Fig. 4d).

Population decoding (Fig. 5): For each unit in a given population, we generated estimates of the target value on each trial based on the minimum spike-distance from templates generated on all other trials (i.e., the same LOCV method as for individual unit decoding—see above). Templates were generated for each unit using neural activity within a 100 ms roving time window. In addition to an estimated target value, we also retained a confidence score for that estimate: the spike-distance from test trial to the closest template, expressed as a proportion of the sum of spike distances between test trial and all templates. Across the population, we then summed confidence weights for each possible feature value and selected the value with the largest sum as the population estimate for that trial. We then repeated the procedure across trials to get the decoding performance of a given population.

We compared the timing of population decoding by calculating the time at which each neural population decoded vowel identity best. This measurement was performed for every population, of every population size (i.e., 1 to 74 units)—shown in the scatter plots in Fig. 5d. Timing of vowel decoding was then compared for sounds varied across orthogonal dimensions using a permutation test: For each orthogonal dimension, we calculated the mean time across populations that gave best decoding performance. We then used the difference between means as the measured variable (i.e., difference between F0 and voicing). We then randomly shuffled the orthogonal dimensions that each population was drawn from and recalculated the difference in mean timing on 10^4^ iterations.

Error trial analysis (Supplementary Fig. 12): We trained the decoder on correct trials using the LOCV procedure to estimate vowel identity on each individual correct trial from templates built on all other correct trials. For error trials, we used the training templates calculated across all correct trials and estimated vowel identity on each error trial. Only units that were informative about vowel identity were analyzed, with the exception of three units recorded when the animal performed perfectly (i.e., made no errors) when vowels varied across sound location and thus error trials could not be studied. We repeated the same procedure for decoding orthogonal variables using only units informative about the relevant dimension. Decoding performance was compared for vowel identity, orthogonal values and for behavioral choice using a Wilcoxon sign-rank test. We compared the change in decoding performance between correct and error trials when decoding vowel identity and behavioral choice using a Wilcoxon rank-sum test.

### Code availability

Custom-written computer code for behavioral and neural data collection and analysis is available from the authors on request.

## Electronic supplementary material

Supplementary Information

## Data Availability

The datasets generated during and/or analyzed in the current study are available from the corresponding authors on reasonable request. Data presented in all figures are available from figshare with the identifier 10.6084/m9.figshare.7176470 [10.6084/m9.figshare.7176470]
