# Supplementary material for: Sound identity is represented robustly in auditory cortex during perceptual constancy
Source: Nat Commun. 2018 Nov 14;9:4786. doi: 10.1038/s41467-018-07237-3 (PMC6235866; doi:10.1038/s41467-018-07237-3)
Supplement: Supplementary file 1 — Supplementary Information [file 41467_2018_7237_MOESM1_ESM.pdf]

Supplementary Information

Sound identity is represented robustly in auditory cortex during perceptual constancy

Town et al.

Supplemental Figures

Supplementary Figure 1 Discrimination of a single vowel token

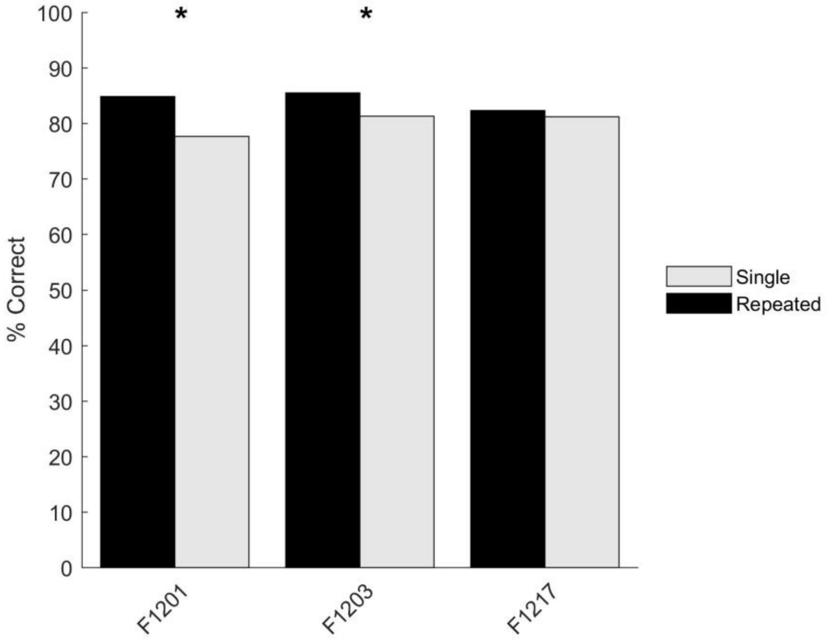

Performance of three subjects tested with a single vowel token (250 ms) that was roved across F0 as described in the main text. Asterisks indicate significant contribution of token number to logistic regression models of each subject’s performance (analysis of deviance, $p < 0.05$).

Supplementary Figure 2 Generalization vs. Memorization

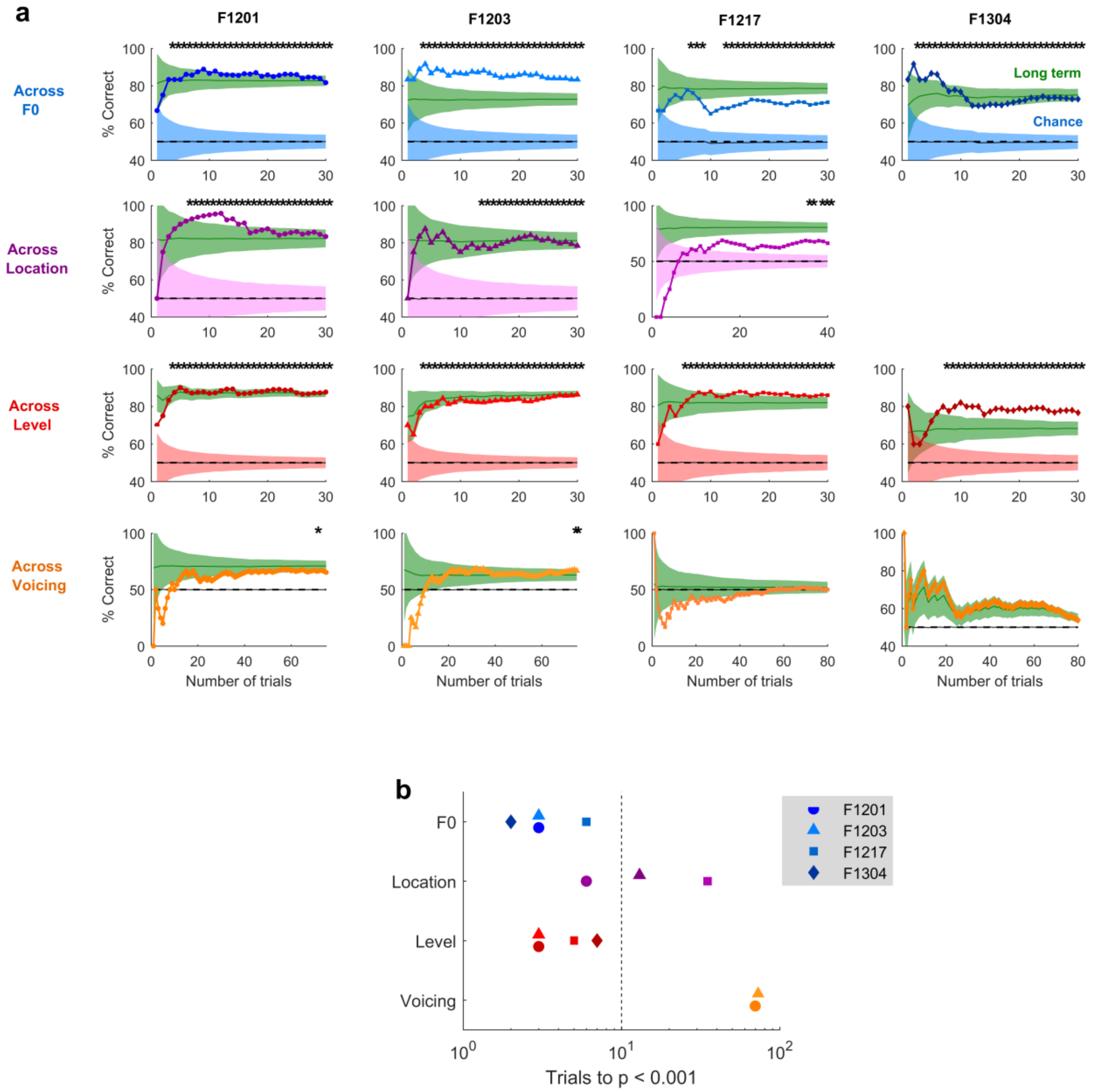

a If animals generalized from the initial training conditions to other orthogonal values, task performance should be better than chance without extended experience. Each ferret's performance was computed in windows beginning with the first trial experienced, and extending out to consider progressively longer durations (F1201: circles; F1203: triangles; F1217: squares; F1304: diamonds). Performance was compared with chance by randomizing the required response across trials and recalculating percent correct (blue, purple and red zones centered on 50% show the mean ± standard deviation across 10^4 iterations). Asterisks show windows for which the probability of observed performance (p) was below 0.001 (permutation test; uncorrected for multiple comparisons). To visualize the effect of trial number on ability to detect successful long-term task performance, we also randomized the trial indices across all available trials (green patches, 400 iterations, data are shown as mean ± standard deviation). **b** Minimum number of trials required for performance to exceed chance performance with a probability less than 0.001 (permutation test) for each orthogonal dimension and subject. Markers as in **a**. Note that performance was never significantly above chance for two ferrets discriminating vowels across voicing conditions.

Supplementary Figure 3 Decoder structure

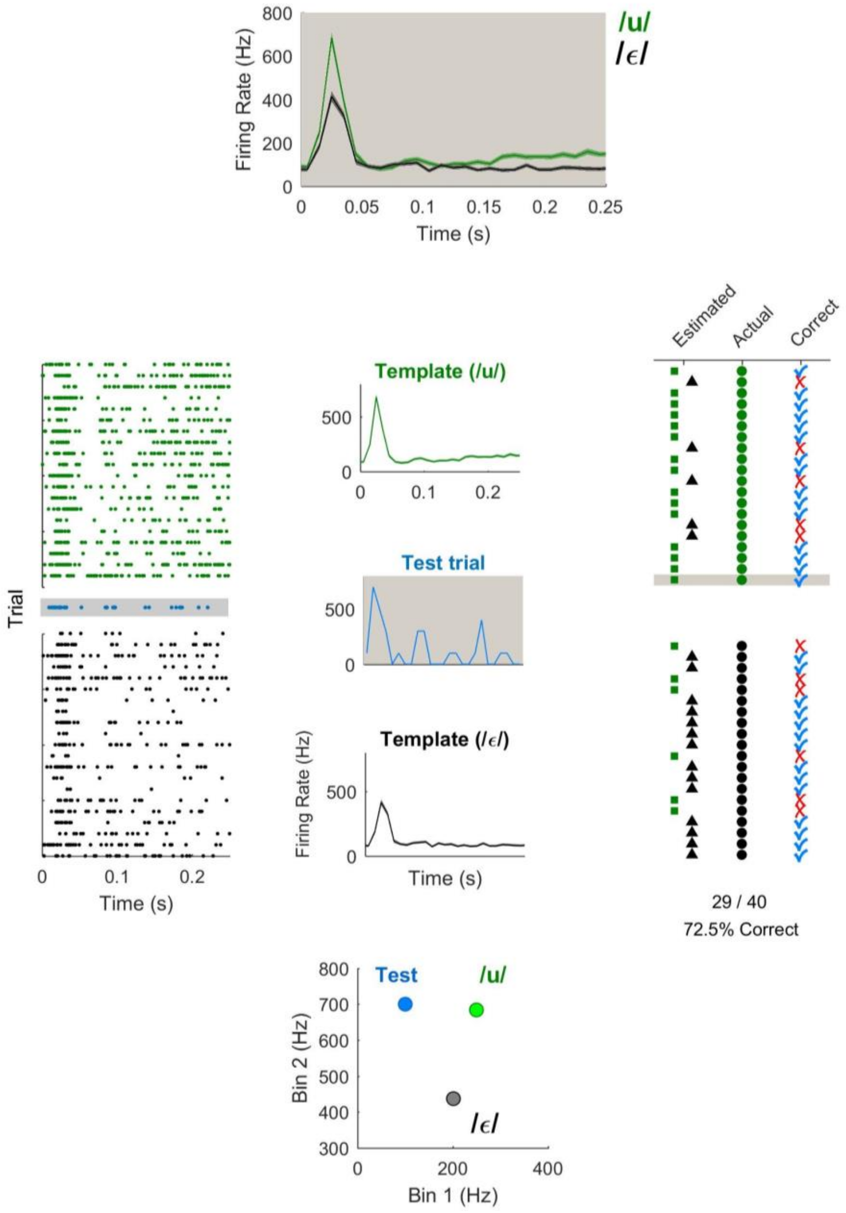

Schematic showing the decoding of trial parameters (e.g. vowel identity, /u/: black; /ε/: green) from single trial neural responses for one unit. We used a leave-one-out cross validation method in which templates were calculated as the mean response to each stimulus class (e.g. vowel) on all but one test trial of the data set. Mean responses were averaged across trials from spike times within a decoding window binned at 10 ms intervals. For the test trial, the decoded estimate of stimulus class was assigned as the template class with the smallest Euclidean distance to the test response. Every trial in the dataset was decoded as a test trial with templates recalculated from all other trials.

Supplementary Figure 4 Optimizing timing parameters

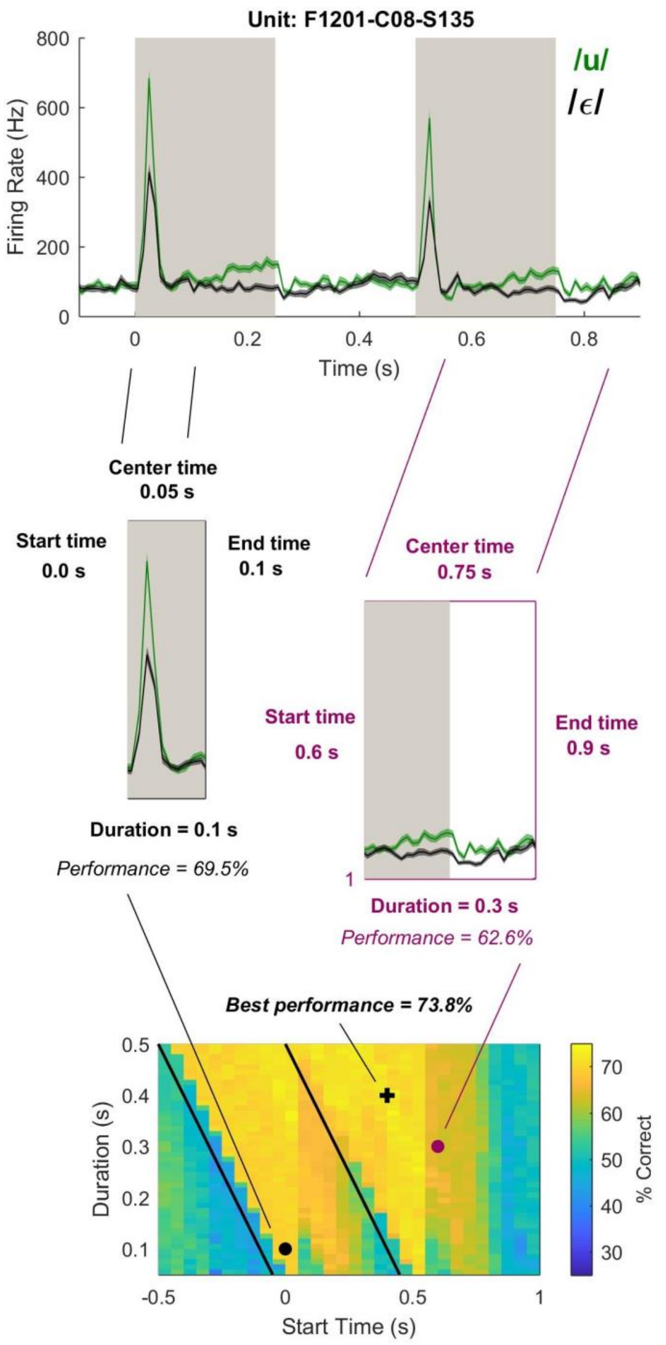

To accommodate potential variation in timing of information content, we varied the temporal parameters (start time and duration) that defined the decoding window. Start time was varied from -0.5 to 1 s after stimulus onset in 50 ms intervals; duration was varied between 10 and 500 ms in 10 ms intervals. For each combination of start time and duration, we calculated decoding performance across trials and mapped temporal parameter space using a simple grid search. (Black diagonal lines across this space represent sound onset times). While this search protocol may not find the true optimized parameters for best decoding performance, it nonetheless enabled us to improve decoding performance and estimate those times in the trial at which information about a given feature was most strongly represented.

Supplementary Figure 5 Permutation testing of decoder performance

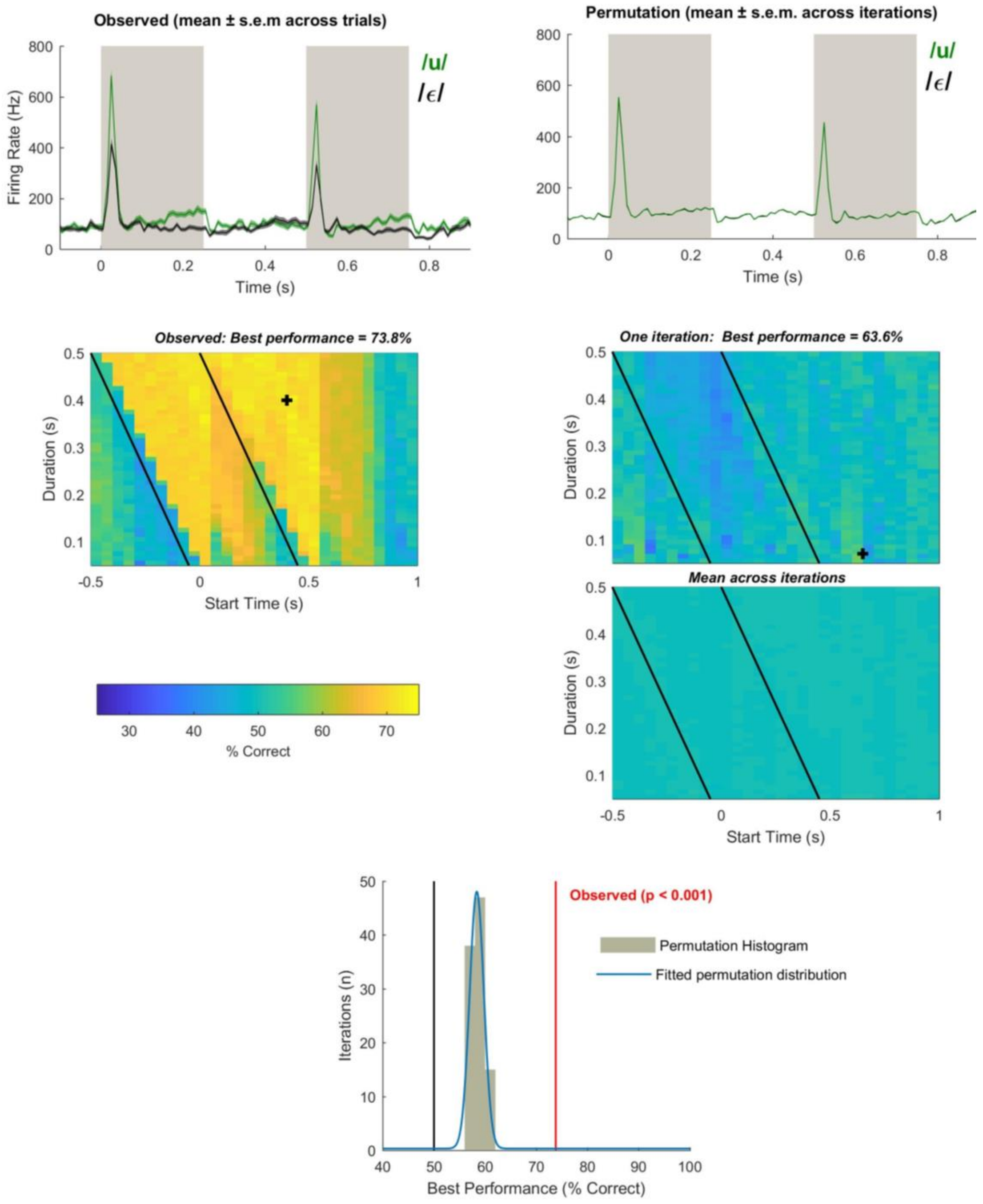

Each test variable (e.g. vowel identity, /u/: black; /ε/: green) was shuffled and the decoding procedure was repeated with the full optimization search. For each unit, we repeated this shuffling procedure on 100 iterations (we used a relatively small number of iterations and parameterized the permutation distribution to compromise for the computational cost of optimization). When shuffled, PSTH responses to each vowel were virtually identical. To determine whether a unit was informative, we fitted a Gaussian function to the distribution of best performance values obtained for each shuffle and calculated the probability of randomly obtaining the observed decoding performance (p). The shuffled distribution shown here was centered above chance performance because of the optimization routine in the decoder, which selected the best performing noise.

Supplementary Figure 6 Improvement in decoding performance with optimization of time window parameters

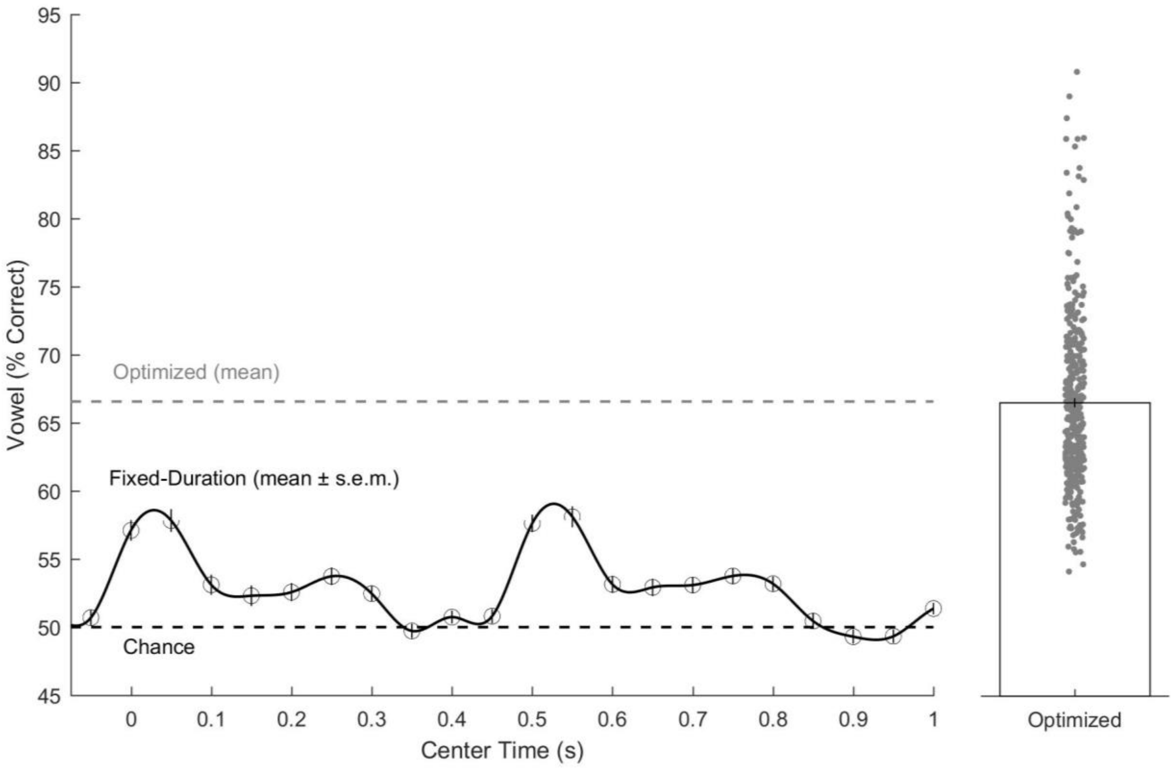

Comparison of performance decoding vowel identity using neural responses in a fixed duration (100 ms centered at different times after stimulus onset) or using optimized time window. Data show mean ± s.e.m. across all units (n = 366), with individual data points showing individual units for optimized data. For each time point, optimized decoding performance was significantly better than fixed window performance (Sign-rank test, Bonferroni corrected for multiple comparisons, $p < 1 \times 10^{-10}$).

Supplementary Figure 7 Conservation of function across multiple orthogonal dimensions

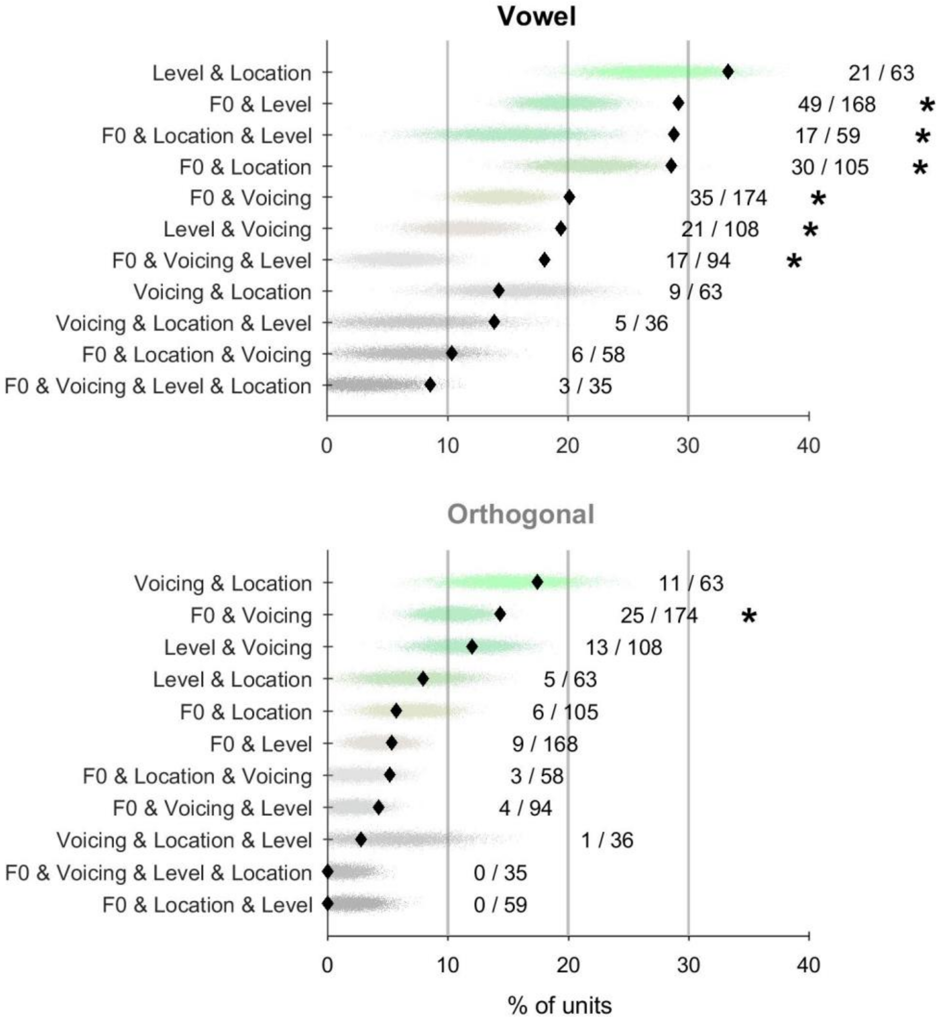

To test if information about sound identity was preserved over multiple orthogonal variables (e.g. sound location and level), we counted the number of units classified as vowel informative across multiple orthogonal dimensions (top)(diamond markers) or about multiple orthogonal dimensions (bottom). The proportion of conserved units was compared to permutation distributions (scatter plots) resulting from random shuffle of unit identity. Asterisks indicate comparisons in which the percentage of conserved units was greater than chance (permutation test, Bonferroni corrected, $p < 0.0045$; Supplementary Table 3). Fractions indicate the number of conserved units as a proportion of units tested (not all units were tested with sounds varying across every orthogonal dimension).

Supplementary Figure 8 Decoding vowel identity at each orthogonal value

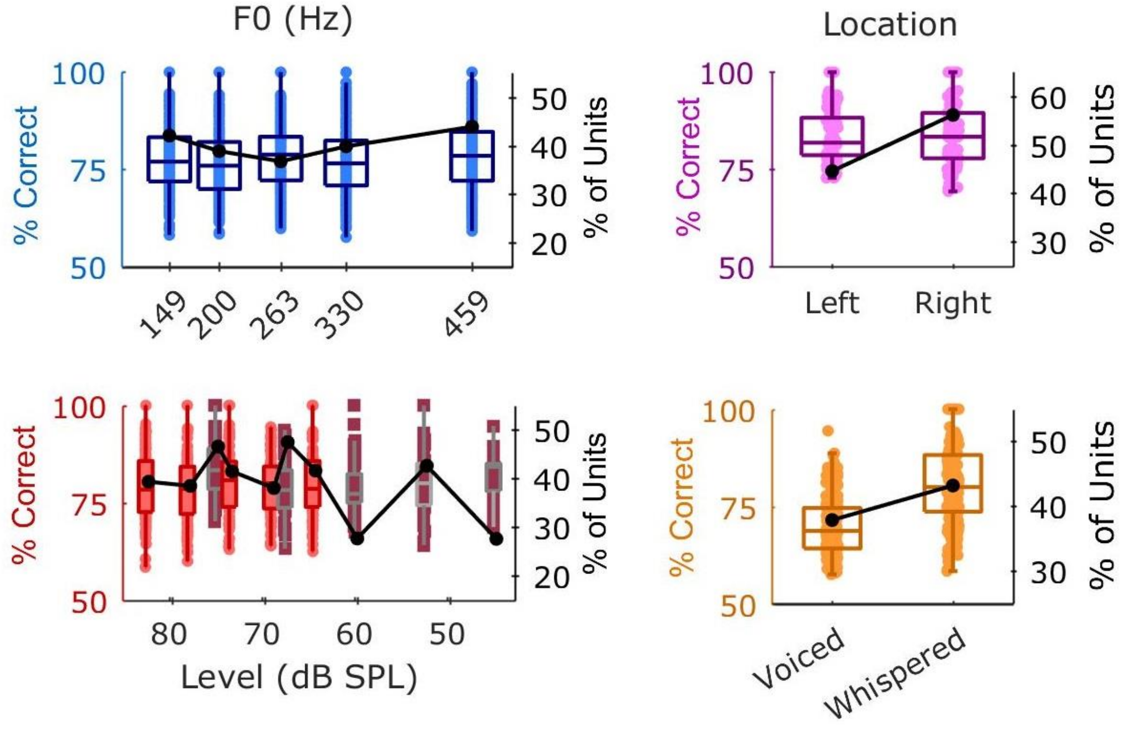

To facilitate direct comparison of decoding and behavioral performance, we decoded vowel identity (boxplots; blue, red, orange and magenta) and calculated the proportion of vowel informative units at each orthogonal value (line plots; black). For sound level, different shades reflect the distinct sound ranges over which units were tested. Decoding performance is shown as boxplots with median, interquartile range (box) and 99.3% intervals (whiskers). Individual data points show performance for each unit. When decoding vowel identity at each orthogonal value, the proportion of vowel informative units was independent of F0 (Blue: logistic regression, χ^2 =0.849, p = 0.357), sound location (Magenta: χ^2 =1.72, p = 0.189) or level (Red: χ^2 = 0.384, p = 0.536). In contrast, ksignificantly fewer units (29.4%; 70/238) were informative about vowel identity when voiced than whispered vowels (Orange: χ^2 = 4.79, p = 0.028).

Supplementary Figure 9 Temporal profiles for the onset (start time) of best decoding windows

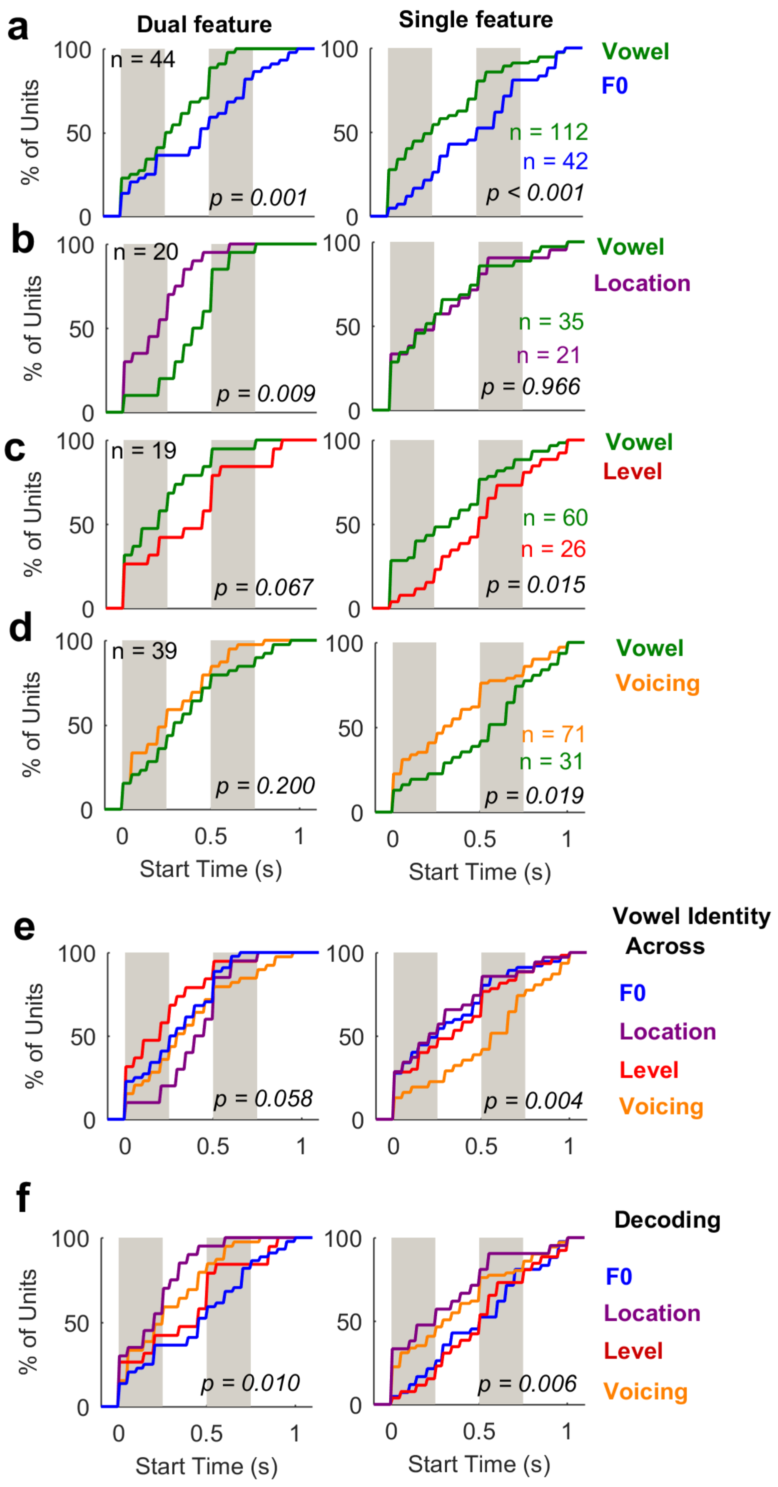

a-d Cumulative distribution functions (CDFs) showing start time for best performance when decoding vowel identity (green) or orthogonal variables (**a:** F0, blue; **b**: Location, purple; **c**: Level, red; **d**: Voicing, orange). Units are shown separately by classification as informative about vowel identity and orthogonal values (Dual feature units), or only vowel identity or orthogonal values

(Single feature units). **e** CDFs for decoding vowel identity across each orthogonal variable. **f** CDFs for decoding orthogonal values across vowels. Data are the same as in **a**-**d** but replotted (and in the case of Vowel, recolored) across orthogonal dimensions (F0: blue; Location: purple; Level: red; Voicing: orange). Values show sample sizes (n) and comparisons (p) between vowel and orthogonal (a-d: rank-sum or sign-rank depending on pairing) or across orthogonal variables (e, f: Kruskal-Wallis test).

Supplementary Figure 10 Temporal profiles for the duration of best decoding windows

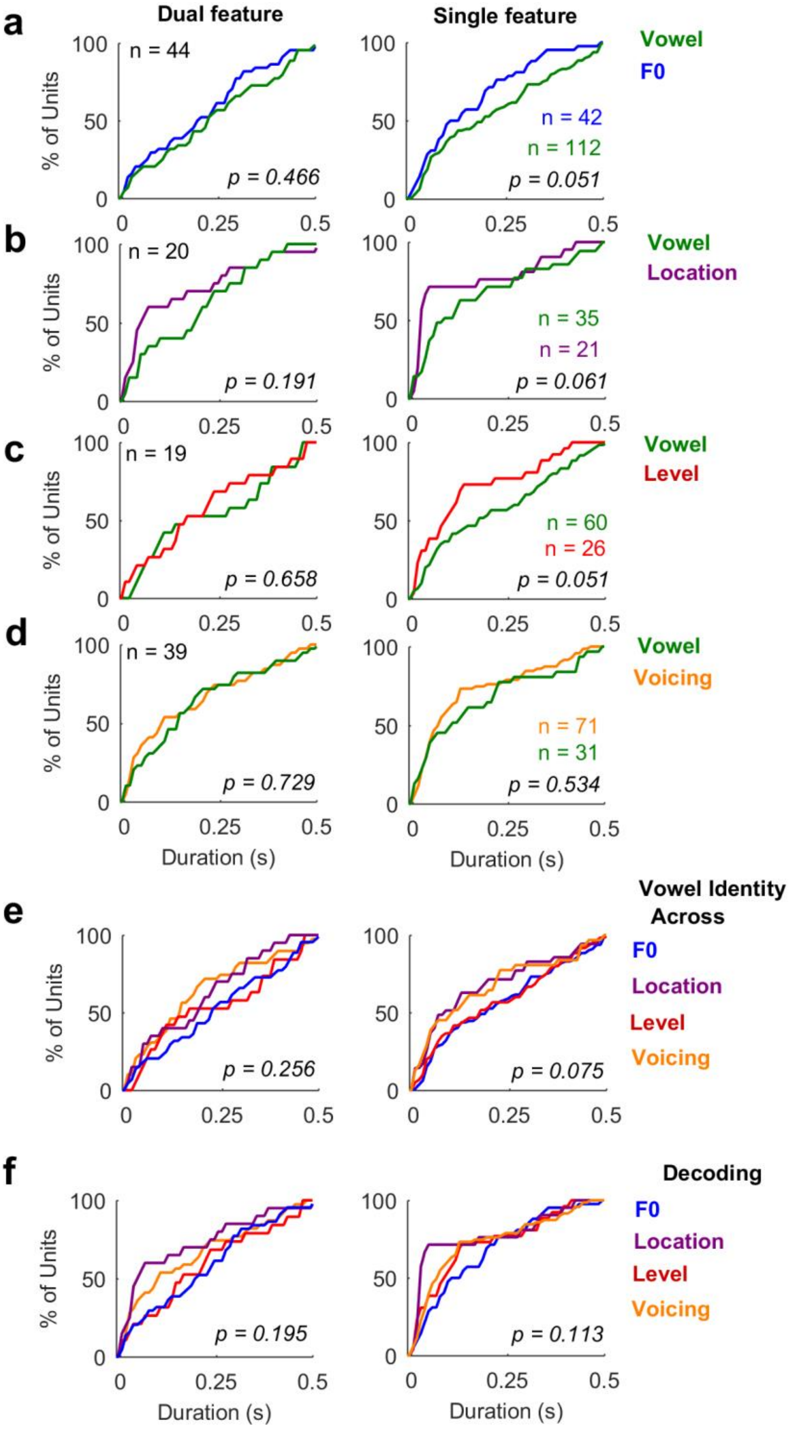

a-d Cumulative distribution functions (CDFs) showing duration for best performance when decoding vowel identity or orthogonal variables (**a:** F0, blue; **b**: Location, purple; **c**: Level, red; **d**: Voicing, orange). Units are shown separately by classification as informative about vowel identity and orthogonal values (Dual feature units), or only vowel identity or orthogonal values (Single feature units). **e** CDFs for decoding vowel identity across each orthogonal variable. **f** CDFs for decoding orthogonal values across vowels. Data are the same as in **a**-**d** but replotted (and in the case of

Vowel, recolored) across orthogonal dimensions (F0: blue; Location: purple; Level: red; Voicing: orange). Values show sample sizes (n) and comparisons (p) between vowel and orthogonal (**a**-**d**: rank-sum or sign-rank depending on pairing) or across orthogonal variables (**e**, **f**: Kruskal-Wallis test).

Supplementary Figure 11 Effects of training on spiking activity and decoding performance

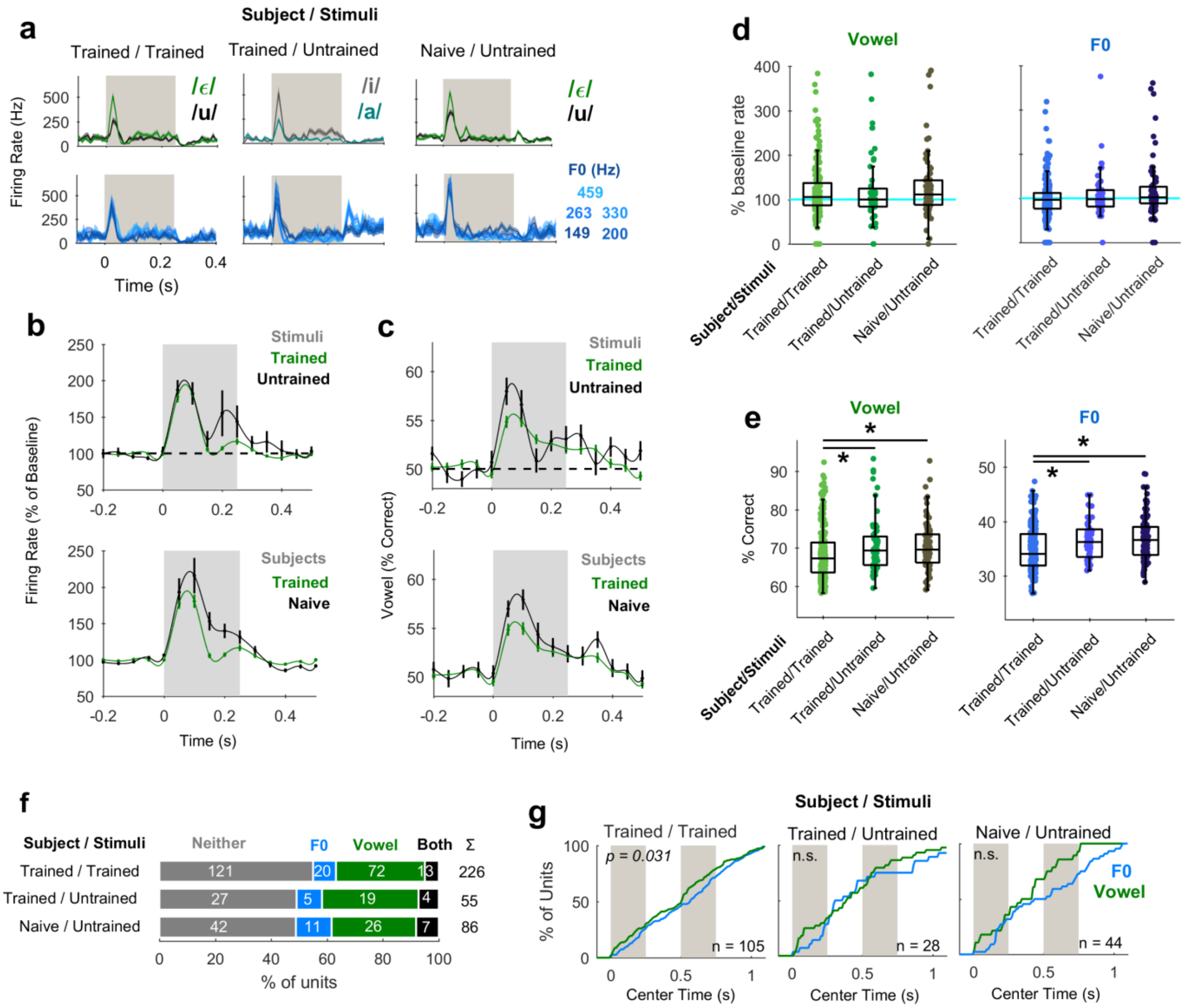

a Example responses to vowels varying in F0 (blue) from individual units recorded during passive listening, from a trained animals presented with trained sounds (left; /u/: black; /ɛ/: green), untrained sounds (middle; /a/: cyan; /i/: grey) or from an untrained animal (right; /u/: black; /ɛ/: green). Data show mean ± s.e.m. firing rates across trials (sample sizes listed in Supplementary Table 6). **b** Population firing rates of all units (Trained subjects tested with trained stimuli: green, n = 226, Trained subjects tested with untrained stimuli: black, n = 55; Naive subjects tested with untrained stimuli: black, n = 86). Data are shown as mean ± s.e.m. in 100 ms windows at 50 ms intervals with spline interpolation across means. Grey bar shows time of first sound. **c** Unpaired comparison of performance decoding vowel identity from unit responses to trained and untrained sounds and units recorded in trained and naïve animals. Data as shown in **b**. **d** Comparison of spiking activity in time windows giving optimized performance when decoding vowel (left) or F0 (right). Data are shown normalized to baseline firing rate in a pre-stimulus baseline period in the 450 ms before stimulus

onset, with scatter plots showing individual units; box plots show median, inter-quartile range (box) and 99.3% intervals (whiskers). Asterisks show significant comparisons between experimental groups (Tukey correction for multiple comparisons, $p < 0.05$). **e** Optimized performance decoding vowel identity (left) or F0 (right) for all units; data is shown as in **d**. Decoding performance differed significantly between experimental groups (Kruskal-Wallis test, $\chi^2 = 12.08$, $p = 0.002$): Decoding of responses to trained sounds was worse than untrained sounds (Post-hoc pairwise comparisons, Tukey-Kramer corrected, $p = 0.046$), and decoding of responses to the same stimuli was worse in units recorded from trained than naïve animals ($p = 0.007$). There was no difference between units in trained and naïve animals responding to untrained sounds ($p = 0.987$). Performance decoding F0 differed significantly between groups (Kruskal-Wallis test, $\chi^2 = 21.0$, $p = 2.76 \times 10^{-5}$) with decoding being worse in units recorded from trained than naïve animals (Post-hoc pairwise comparisons, Tukey-Kramer corrected, $p = 1.0 \times 10^{-4}$), and worse for units responding to trained than untrained sounds ($p = 0.007$). Performance decoding F0 of untrained sounds in naïve and trained animals was not significantly different ($p = 0.935$). **f** Proportion of units classified as informative about vowel and/or F0 in each condition. **g** Cumulative density functions showing the center time of the decoding window giving earliest best performance for vowel identity (green) and F0 (blue). In all conditions, decoding vowel preceded F0; however only for units recorded in trained animals in response to trained sounds was the difference significant. Values (p) show comparison of time at which vowel identity and F0 were best decoded (sign-rank test).

Supplementary Figure 12 Decoding performance on correct and incorrect trials

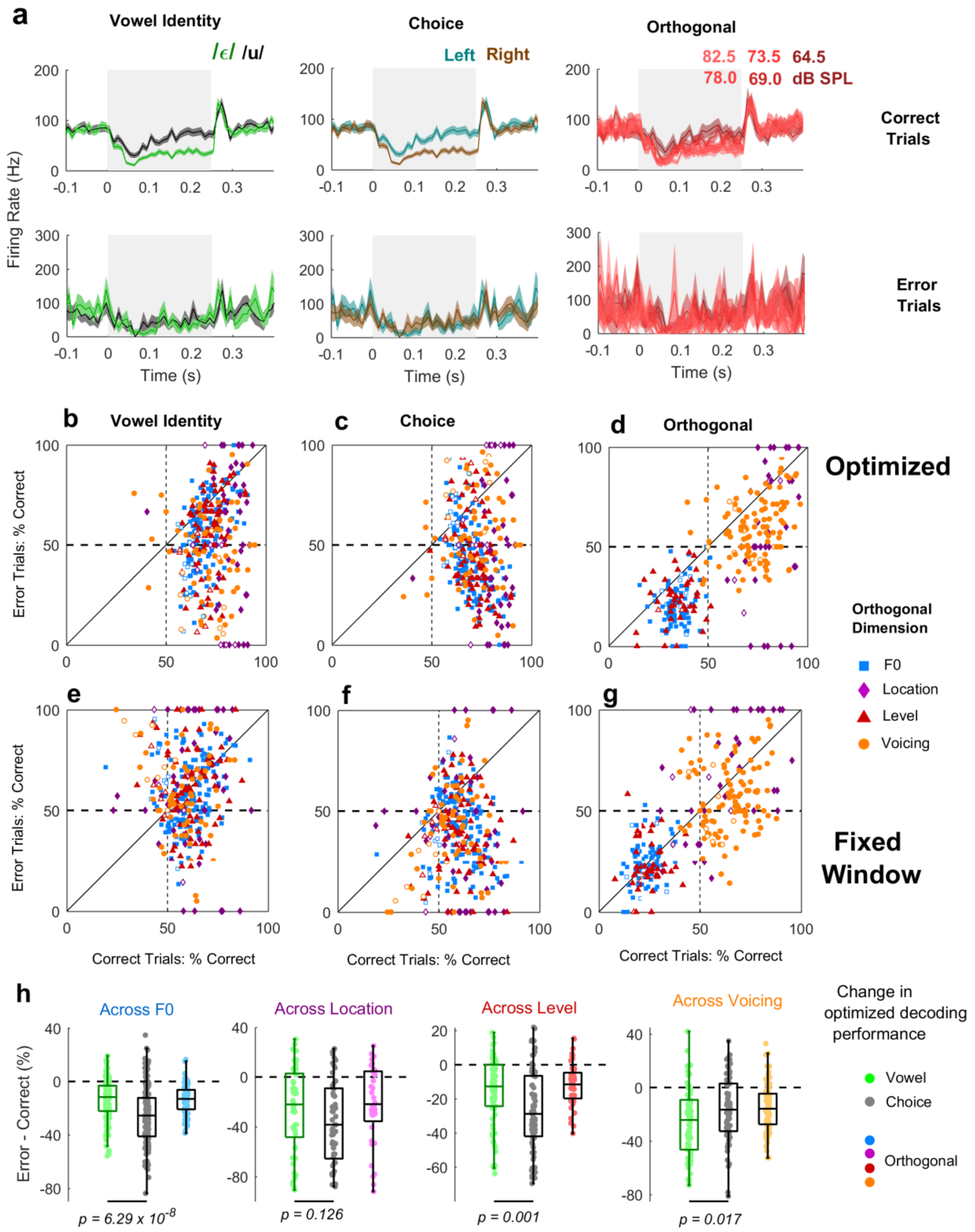

a Discrimination of vowel identity (/u/: black; /ɛ/: green), behavioral choice (left: cyan; right: brown) and sound level (red) by an example unit on correct and error trials. Background bars represent the

duration of the first vowel token after stimulus onset; neural responses shown as mean ± s.e.m. firing rates across trials (sample sizes listed in Supplementary Table 6). **b-g** Performance of all units when decoding vowel identity, choice or orthogonal features on correct and error trials for all units using decoders with optimized (**b-d**) or fixed (**e-g**; 0 to 250 ms) time windows. Data presented separately for vowels varied across F0 (blue squares), location (purple diamonds), sound level (red triangles) and voicing (orange circles). Single/multi-unit data shown by unfilled/filled markers. Performance was significantly worse on error trials than correct trials for all comparisons of optimized decoders, but not when fixed time windows were used (Sign-rank test, $p < 0.001$, Supplementary Table 7). **h** Comparison of the error-related decline on decoding vowel identity (green), behavioral choice (grey) and orthogonal features (blue, purple, red and orange). Data is shown for performance of optimized decoders; box plots show median, inter-quartile range (box) and 99.3% intervals (whiskers). Values (p) show that, for sounds varied across F0, sound level or location, error-related decline in performance was greater when decoding behavioral choice – although only significantly so for sound level and F0 (Rank-sum test, $p \leq 0.001$, Supplementary Table 5). In contrast, error-related decline in performance was greater when decoding sound identity than behavioral choice (Supplementary Table 8).

Supplemental Tables

Supplementary Table 1 Comparison of observed vowel discrimination against chance performance

		Ferret			
**Dimension**	**Data Set**	**F1201**	**F1203**	**F1217**	**F1304**
F0	All	10214 / 12036<br>84.9%<br>$p < 0.001$	8502 / 9947<br>85.5%<br>$p < 0.001$	3946 / 4792<br>82.4%<br>$p < 0.001$	1051 / 1487<br>70.7%<br>$p < 0.001$
Location	All	701 / 879<br>79.8%<br>$p < 0.001$	564 / 703<br>80.2%<br>$p < 0.001$	112 / 145<br>77.2%<br>$p < 0.001$	Not tested
Sound level (dB SPL)	45	138 / 186<br>74.2%<br>$p < 0.001$	149 / 196<br>76.0%<br>$p < 0.001$	Not tested	Not tested
	52.5	151 / 193<br>78.2%<br>$p < 0.001$	169 / 205<br>82.4%<br>$p < 0.001$	Not tested	Not tested
	60	161 / 192<br>83.9%<br>$p < 0.001$	162 / 188<br>86.2%<br>$p < 0.001$	Not tested	Not tested
	64.5	438 / 498<br>88.0%<br>$p < 0.001$	372 / 414<br>89.9%<br>$p < 0.001$	341 / 442<br>77.2%<br>$p < 0.001$	Not tested
	67.5	175 / 195<br>89.7%<br>$p < 0.001$	176 / 198<br>88.9%<br>$p < 0.001$	Not tested	Not tested
	69	479 / 523<br>91.6%<br>$p < 0.001$	357 / 397<br>89.9%<br>$p < 0.001$	345 / 449<br>76.8%<br>$p < 0.001$	Not tested
	73.5	460 / 515<br>89.3%<br>$p < 0.001$	352 / 407<br>86.5%<br>$p < 0.001$	380 / 457<br>83.2%<br>$p < 0.001$	Not tested
	75	168 / 187<br>89.8%<br>$p < 0.001$	179 / 197<br>90.9%<br>$p < 0.001$	Not tested	Not tested
	78	445 / 501<br>88.8%<br>$p < 0.001$	382 / 434<br>88.0%<br>$p < 0.001$	392 / 442<br>88.7%<br>$p < 0.001$	Not tested
	82.5	445 / 500<br>89.0%<br>$p < 0.001$	357 / 424<br>84.2%<br>$p < 0.001$	366 / 424<br>86.3%<br>$p < 0.001$	Not tested
	All	Not tested	Not tested	Not tested	309 / 455<br>67.9%<br>$p < 0.001$
Voicing	Voiced	3830 / 4472<br>85.6%<br>$p < 0.001$	2833 / 3207<br>88.3%<br>$p < 0.001$	2585 / 3038<br>85.1%<br>$p < 0.001$	333 / 473<br>70.4%<br>$p < 0.001$
	Whispered	870 / 1332<br>65.3%<br>$p < 0.001$	596 / 1007<br>59.2%<br>$p < 0.001$	386 / 748<br>51.6%<br>$p = 0.400$	83 / 146<br>56.9%<br>$p = 0.116$

Data shown as fraction of trials correct and probability of observed performance (Binomial test vs. 50%). Orthogonal values tested separately for sound level and voicing, when a significant main effect of orthogonal value was observed on behavioral performance (Supplementary Table 2).

Supplementary Table 2 Logistic regressions comparing behavioral performance across orthogonal variables

	Orthogonal Dimension			
**Ferret**	**F0 (149, 200, 263, 330 & 459 Hz)**	**Location (±90°) (i.e. Left / Right)**	**Sound Level (45 – 82.5 dB SPL)**	**Voicing (Voiced / Whispered)**
F1201	df = 12034, p = 0.7731	df = 879, p = 0.219	df = 3490, p < 0.001	df = 5802, p < 0.001
F1203	df = 9945, p = 0.764	df = 701, p = 0.185	df = 3063, p = 0.005	df = 4212, p < 0.001
F1217	df = 4790, p = 0.368	df = 145, p = 0.523	df = 2214, p < 0.001	df = 3784, p < 0.001
F1304	df = 1485, p = 0.388	Not tested	df = 455, p = 0.882	df = 617, p = 0.002

Supplementary Table 3 Permutation tests of conserved classification

a

Orthogonal dimensions	Dimensions (n)	Conserved (n)	Total (n)	p
Level & Location	2	21	63	0.018
F0 & Level	2	49	168	<0.001
F0 & Location & Level	3	17	59	<0.001
F0 & Location	2	30	105	0.001
F0 & Voicing	2	35	174	<0.001
Level & Voicing	2	21	108	<0.001
F0 & Voicing & Level	3	17	94	<0.001
Voicing & Location	2	9	63	0.570
Voicing & Location & Level	3	5	36	0.017
F0 & Location & Voicing	3	6	58	0.067
F0 & Voicing & Level & Location	4	3	35	0.007

b

Orthogonal dimensions	Dimensions (n)	Conserved (n)	Total (n)	p
Voicing & Location	2	11	63	0.150
F0 & Voicing	2	25	174	0.003
Level & Voicing	2	13	108	0.326
Level & Location	2	5	63	0.261
F0 & Location	2	6	105	0.618
F0 & Level	2	9	168	0.172
F0 & Location & Voicing	3	3	58	0.032
F0 & Voicing & Level	3	4	94	0.031
Voicing & Location & Level	3	1	36	0.587
F0 & Voicing & Level& Location	4	0	35	0.232
F0 & Location & Level	3	0	59	0.671

a Permutation test results (p) when asking if populations of units with conserved classification as vowel informative were larger than chance (Supplementary Figure 7). Tests used 10^4 iterations. Population size was described as significant using $\alpha = 0.0045$ (Bonferroni corrected for 11 comparisons). **b** Permutation test for units with conserved classification as informative about multiple orthogonal dimensions.

Supplementary Table 4 Breakdown of units by single and multi-unit status

Orthogonal Dimension	Vowel informative		Orthogonal Informative		Total	
	SU	MU	SU	MU	SU	MU
F0	14 (18.7%)	142 (41.0%)	10 (13.3%)	76 (22.0%)	75	346
Location	9 (27.3%)	46 (45.1%)	5 (15.2%)	36 (35.3%)	33	102
Level	6 (17.1%)	73 (39.2%)	3 (8.57%)	42 (22.6%)	35	186
Voicing	9 (20.9%)	61 (31.3%)	9 (20.9%)	101 (51.8%)	43	195

Supplementary Table 5 Timing differences decoding orthogonal dimensions

Orthogonal Features		Dual Feature	Single Feature
F0	Location	p = 0.001	p = 0.022
	Level	n.s.	n.s.
	Voicing	p = 0.022	n.s.
Location	Level	n.s.	p = 0.043
	Voicing	n.s.	n.s.
Level	Voicing	n.s.	n.s.

Pairwise comparison of timing differences when decoding orthogonal dimensions. Comparison of decoding times across stimulus features also demonstrated significant differences between orthogonal variables, both for dual-feature (Kruskal-Wallis test, $\chi^2 = 16.36$, $p = 0.001$) and single feature units (Kruskal-Wallis test, $\chi^2 = 13.22$, $p = 0.004$). Table values give pair-wise comparisons with Tukey correction.

Supplementary Table 6 Sample sizes for example peri-stimulus time histograms (PSTHs) throughout the study

Figure	Condition	Trials (n)	Condition	Trials (n)
**3a** (F1203 C29 S32)	Engaged, /u/	94	Passive, /u/	31
	Engaged, /ɛ/	124	Passive, /ɛ/	33
	Engaged, F0 = 149 Hz	45	Passive, F0 = 149 Hz	14
	Engaged, F0 = 200 Hz	41	Passive, F0 = 200 Hz	12
	Engaged, F0 = 263 Hz	46	Passive, F0 = 263 Hz	13
	Engaged, F0 = 330 Hz	39	Passive, F0 = 330 Hz	10
	Engaged, F0 = 459 Hz	47	Passive, F0 = 459 Hz	15
**S11a** (F1203 C29 S32)	Trained/Trained, /u/	31	Trained/Untrained, /i/	19
	Trained/Trained, /ɛ/	33	Trained/Untrained, /a/	31
	Trained/Trained, F0 = 149 Hz	14	Trained/Untrained, F0 = 149 Hz	10
	Trained/Trained, F0 = 200 Hz	12	Trained/Untrained, F0 = 200 Hz	10
	Trained/Trained, F0 = 263 Hz	13	Trained/Untrained, F0 = 263 Hz	13
	Trained/Trained, F0 = 330 Hz	10	Trained/Untrained, F0 = 330 Hz	9
	Trained/Trained, F0 = 459 Hz	15	Trained/Untrained, F0 = 459 Hz	8
**S11a** (F1306 C25 S4)	Naive /Untrained, /u/	33		
	Naive /Untrained, /ɛ/	38	Naive/Untrained, F0 = 263 Hz	14
	Naive/Untrained, F0 = 149 Hz	17	Naive/Untrained, F0 = 330 Hz	15
	Naive /Untrained, F0 = 200 Hz	14	Naive/Untrained, F0 = 459 Hz	11
**S12a** (F1201 C16 S125)	Correct, /u/	168	Error, /u/	18
	Correct, /ɛ/	190	Error, /ɛ/	15
	Correct, Left	168	Error, Left	18
	Correct, Right	190	Error, Right	15
	Correct, 64.5 dB SPL	64	Error, 64.5 dB SPL	10
	Correct, 69.0 dB SPL	79	Error, 69.0 dB SPL	6
	Correct, 73.5 dB SPL	74	Error, 73.5 dB SPL	8
	Correct, 78.0 dB SPL	72	Error, 78.0 dB SPL	4
	Correct, 82.5 dB SPL	69	Error, 82.5 dB SPL	5

Supplementary Table 7 Comparisons of decoding on correct and error trials

Optimized		Vowel: [u] vs [ε] (Supplementary Figure 12b)		Choice: Left vs. Right (Supplementary Figure 12c)		Orthogonal (Supplementary Figure 12d)	
		z	p	z	p	z	P
Orthogonal Dimension	F0	-8.82	1.16×10^{-18}	-9.69	3.24×10^{-22}	-7.37	1.66×10^{-13}
	Location	-4.36	1.27×10^{-5}	-5.40	6.82×10^{-8}	-3.59	3.32×10^{-4}
	Level	-5.92	3.25×10^{-9}	-6.66	2.63×10^{-11}	-4.62	3.79×10^{-6}
	Voicing	-6.21	5.43×10^{-10}	-4.71	2.5×10^{-6}	-7.30	2.92×10^{-13}

(Column group header: Decoded Feature)

Fixed Time Window		Vowel: [u] vs [ε] (Supplementary Figure 12e)		Choice: Left vs. Right (Supplementary Figure 12f)		Orthogonal (Supplementary Figure 12g)	
		z	p	z	p	z	p
Orthogonal Dimension	F0	-1.95	0.051	-8.56	1.11×10^{-17}	0.691	0.489
	Location	0.376	0.707	-3.94	8.01×10^{-5}	0.968	0.333
	Level	0.010	0.992	-6.38	1.74×10^{-10}	-0.969	0.333
	Voicing	0.975	0.330	-5.18	2.21×10^{-7}	-4.27	1.93×10^{-5}

Comparisons of decoding performance on correct and error trials (sign-rank test vs. no change) using optimized time windows (Supplementary Figure 12). All comparisons of vowel and choice decoding were made using units that were classed as significantly vowel informative. Comparisons of orthogonal feature decoding were made using only units that were classed as significantly informative about the respective orthogonal dimension.

Supplementary Table 8 Comparisons of change in decoding performance

		Effect of trial correct:			
		**Vowel vs. Choice**		**Orthogonal vs. Choice**	
		z	p	z	p
Orthogonal Dimension	F0	5.14	6.29×10^{-8}	4.76	1.95×10^{-6}
	Location	1.53	0.126	2.02	0.044
	Level	3.26	0.001	3.82	1.34×10^{-4}
	Voicing	-2.39	0.017	0.23	0.816

Comparisons of *change* in performance decoding vowel identity and behavioral choice on error trials (sign-rank test vs. no change) (Supplementary Figure 12). Note that for sounds varying in voicing, decoding of behavioral choice using optimal time windows was better than vowel identity (hence the change in sign of test statistic, z); whereas for all other comparisons, decoding of choice was worse than vowel identity. Mean denotes the mean change (vowel –choice) of decoding performance change between error and correct trials.
